# Epigenomic response to albuterol treatment in asthma-relevant airway epithelial cells

**DOI:** 10.1186/s13148-023-01571-0

**Published:** 2023-10-03

**Authors:** Javier Perez-Garcia, Maria Pino-Yanes, Elizabeth G. Plender, Jamie L. Everman, Celeste Eng, Nathan D. Jackson, Camille M. Moore, Kenneth B. Beckman, Vivian Medina, Sunita Sharma, Daniel Efrain Winnica, Fernando Holguin, José Rodríguez-Santana, Jesús Villar, Elad Ziv, Max A. Seibold, Esteban G. Burchard

**Affiliations:** 1 Genomics and Health Group, Department of Biochemistry, Microbiology, Cell Biology, and Genetics, Universidad de La Laguna (ULL), La Laguna, Tenerife, Canary Islands Spain; 2 CIBER de Enfermedades Respiratorias, Instituto de Salud Carlos III, Madrid, Spain; 3 Instituto de Tecnologías Biomédicas (ITB), Universidad de La Laguna (ULL), La Laguna, Spain; 4 Center for Genes, Environment, and Health, National Jewish Health, Denver, CO USA; 5 Department of Medicine, University of California San Francisco (UCSF), San Francisco, CA USA; 6 Department of Biomedical Research, National Jewish Health, Denver, CO USA; 7 Department of Biostatistics and Informatics, University of Colorado, Denver, CO USA; 8 University of Minnesota Genomics Center (UMNGC), Minneapolis, MN USA; 9 Centro de Neumología Pediátrica, San Juan, PR USA; 10 Division of Pulmonary Sciences and Critical Care Medicine, University of Colorado School of Medicine, Aurora, CO USA; 11 Multidisciplinary Organ Dysfunction Evaluation Research Network (MODERN), Research Unit, Hospital Universitario Dr. Negrín, Las Palmas de Gran Canaria, Spain; 12 Li Ka Shing Knowledge Institute at the St. Michael’s Hospital, Toronto, ON Canada; 13 Institute for Human Genetics, University of California San Francisco (UCSF), San Francisco, CA USA; 14 Department of Epidemiology and Biostatistics, University of California, San Francisco School of Medicine, San Francisco, CA USA; 15 Department of Pediatrics, National Jewish Health, Denver, CO USA; 16 Department of Bioengineering and Therapeutic Sciences, University of California San Francisco (UCSF), San Francisco, CA USA

**Keywords:** Airway cells, Albuterol, β_2_-agonist, *CREB3L1*, DNA methylation, Epigenetics, EWAS, *KSR1*, *MYLK4*, Puerto Ricans

## Abstract

**Background:**

Albuterol is the first-line asthma medication used in diverse populations. Although DNA methylation (DNAm) is an epigenetic mechanism involved in asthma and bronchodilator drug response (BDR), no study has assessed whether albuterol could induce changes in the airway epithelial methylome. We aimed to characterize albuterol-induced DNAm changes in airway epithelial cells, and assess potential functional consequences and the influence of genetic variation and asthma-related clinical variables.

**Results:**

We followed a discovery and validation study design to characterize albuterol-induced DNAm changes in paired airway epithelial cultures stimulated in vitro with albuterol. In the discovery phase, an epigenome-wide association study using paired nasal epithelial cultures from Puerto Rican children (*n* = 97) identified 22 CpGs genome-wide associated with repeated-use albuterol treatment (*p* < 9 × 10^–8^). Albuterol predominantly induced a hypomethylation effect on CpGs captured by the EPIC array across the genome (probability of hypomethylation: 76%, *p* value = 3.3 × 10^–5^). DNAm changes on the CpGs cg23032799 (*CREB3L1*), cg00483640 (*MYLK4-LINC01600*), and cg05673431 (*KSR1*) were validated in nasal epithelia from 10 independent donors (false discovery rate [FDR] < 0.05). The effect on the CpG cg23032799 (*CREB3L1*) was cross-tissue validated in bronchial epithelial cells at nominal level (*p* = 0.030). DNAm changes in these three CpGs were shown to be influenced by three independent genetic variants (FDR < 0.05). In silico analyses showed these polymorphisms regulated gene expression of nearby genes in lungs and/or fibroblasts including *KSR1* and *LINC01600* (6.30 × 10^–14^ ≤ *p* ≤ 6.60 × 10^–5^). Additionally, hypomethylation at the CpGs cg10290200 (*FLNC*) and cg05673431 *(KSR1*) was associated with increased gene expression of the genes where they are located (FDR < 0.05). Furthermore, while the epigenetic effect of albuterol was independent of the asthma status, severity, and use of medication, BDR was nominally associated with the effect on the CpG cg23032799 (*CREB3L1)* (*p* = 0.004). Gene-set enrichment analyses revealed that epigenomic modifications of albuterol could participate in asthma-relevant processes (e.g., IL-2, TNF-α, and NF-κB signaling pathways). Finally, nine differentially methylated regions were associated with albuterol treatment, including *CREB3L1*, *MYLK4*, and *KSR1* (adjusted *p* value < 0.05).

**Conclusions:**

This study revealed evidence of epigenetic modifications induced by albuterol in the mucociliary airway epithelium. The epigenomic response induced by albuterol might have potential clinical implications by affecting biological pathways relevant to asthma.

**Supplementary Information:**

The online version contains supplementary material available at 10.1186/s13148-023-01571-0.

## Background

Asthma is a leading worldwide biomedical concern, affecting up to 300 million people and related to one of each 250 deaths worldwide [1]. Treatment response to current therapies is heterogeneous, with 10% of patients not responding to available therapies in whom severe asthma remains unresolved [1]. It carries a high burden for patients, families, and healthcare systems, since non-responders suffer frequently from asthma exacerbations, have a lower quality of life, and increased mortality rates [1]. The impact of asthma across different regions and populations is not homogeneous [2], and low-income populations, such as Puerto Ricans, have reported the highest asthma prevalence, impaired lung function, and mortality [3]. Albuterol, a short-acting β_2_-agonist bronchodilator, is the most widely used drug to relieve asthma symptoms and the current first-line asthma drug in these populations. However, albuterol effectiveness, measured by the bronchodilator drug response (BDR), is the lowest in African Americans and Puerto Ricans in comparison with other populations such as Mexican Americans [4–6]. Despite this, these populations are prone to use albuterol exclusively in the management of their asthma, rather than more expensive maintenance medications, including inhaled corticosteroids, leading to the overuse of these short-acting bronchodilators. The molecular effects of this overuse on the airway epithelium which lines the airway lumen are unknown.

Genetics and environmental factors contribute to asthma pathophysiology. Since 2007, genome-wide association studies (GWAS) have provided new insights into the genetics of asthma and BDR [4–8]. However, identified genetic variants are unable to explain the heritability of asthma susceptibility and treatment response, estimated at over 60–65% [7, 9]. Epigenetic modifications regulate gene expression without modifying the underlying DNA sequence, being DNA methylation (DNAm) the most widely studied epigenetic marker [10]. Elucidating the role of epigenetic mechanisms in asthma has been of great interest since they might mediate the contribution of genetics and the environment to the disease [7, 10]. In epigenetic studies, nasal samples are one of the most commonly used as non-invasive proxies of the lung environment [11], as the nasal airway transcriptome has been demonstrated to reflect asthma status and is highly correlated with that of the bronchial airway cells [12].

Epigenome-wide association studies (EWAS) have sought epigenetic biomarkers to explain missing asthma heritability [7, 10, 11]. Epigenetics has also been implicated in lung function and albuterol response in children [13–15]. Our group recently identified DNAm markers associated with BDR in African American and Latino children with asthma that interplay with genetic variation and gene expression, with potential applicability to BDR classification [14]. Although epigenetic changes are modifiable, there is a lack of studies evaluating the impact of asthma therapies on DNAm. To our best, few studies have assessed the potential epigenomic response of corticosteroids [16, 17], but none have focused on albuterol.

We hypothesized that albuterol induces genome-wide DNAm changes in the airway epithelium and that repeated exposures to albuterol will durably reprogram the molecular response of these cells. This study aimed to characterize the effect of albuterol on DNAm on the mucociliary airway epithelium, and to assess the influence of genetic variation and asthma-related clinical characteristics (i.e., asthma severity, medication use, and BDR), and potential functional consequences on gene expression and biological pathways. Epigenomic modifications were evaluated in a discovery and validation study design using in vitro models of paired primary nasal mucociliary epithelial cultures exposed or not to repeated doses of albuterol.

## Results

### Airway epithelial model of albuterol treatment

**Fig. 1:**
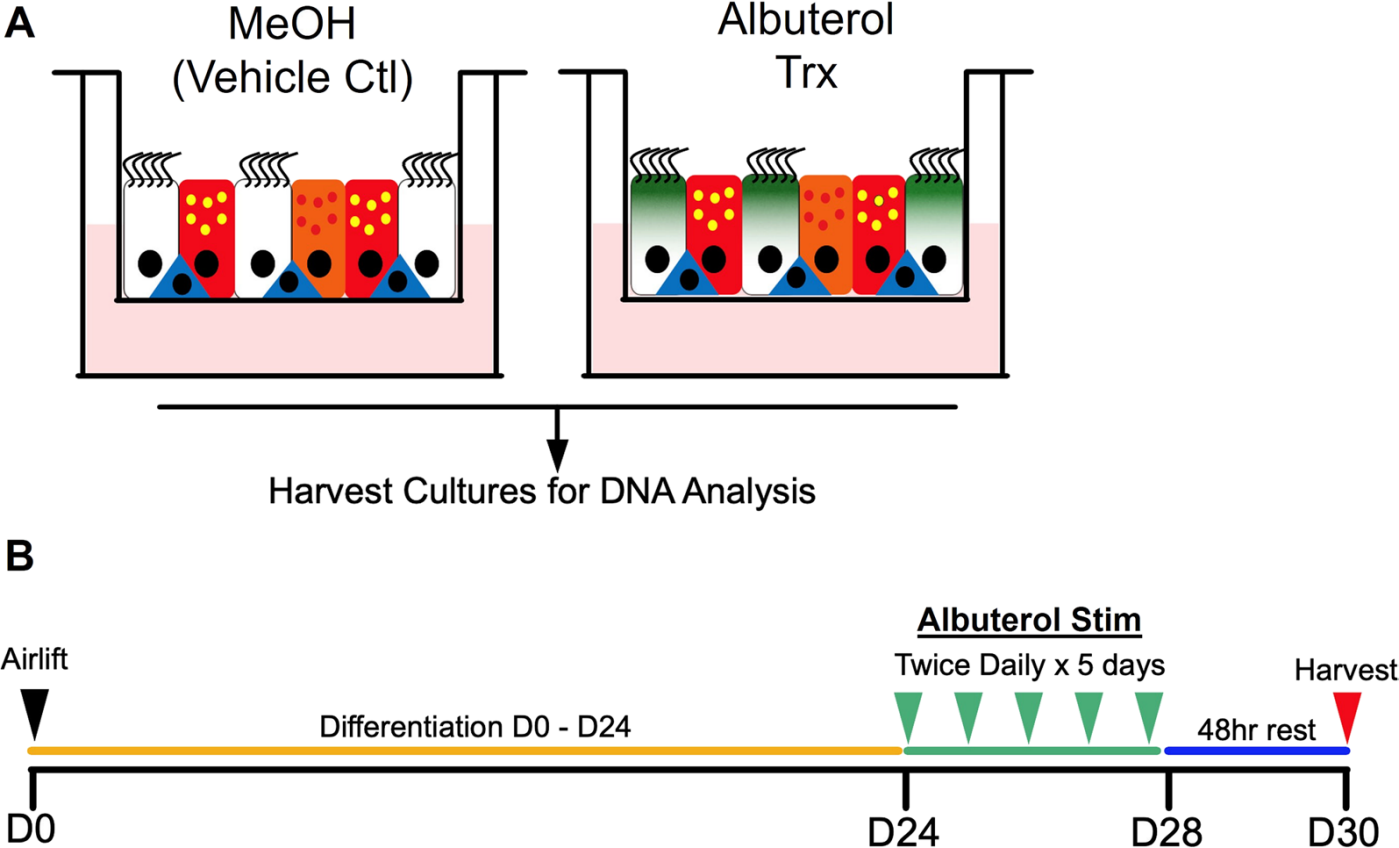
Outline of the laboratory experiments design. **A** Air–liquid interface (ALI) mucociliary airway epithelia were generated from basal stem cells obtained from nasal brushing. Paired samples were stimulated with albuterol (Trx) and vehicle control (MeOH). **B** Experiment timeline. Basal airway epithelial cells were differentiated for 24 days (yellow), followed by 5 days of either control vehicle or albuterol stimulation (green). Cultures were harvested after 48 h of rest from the last stimulation (blue)

To model human airway epithelial molecular responses to albuterol, we established an in vitro nasal airway epithelial culture system. In particular, we generated air–liquid interface (ALI), mucociliary airway epithelia from nasal brushing-derived basal stem cells (Fig. 1). To mimic chronic, repeated albuterol use, we stimulated paired donor cultures with either 100 μM albuterol or mock-stimulus, twice daily, for five consecutive days (Fig. 1). This stimulation course was not associated with visible toxicity (by light microscopy) or loss of epithelial barrier function.

We leveraged our repeated albuterol stimulation model to evaluate epigenomic modifications induced by this treatment in a large cohort of children from the Genes-environments & Admixture in Latino Americans (GALA II) study with nasal airway epithelial brushings. After 48 h post-last exposure to the stimulation course, cultures were harvested for DNA extraction. This timing was selected to allow acute methylation changes induced by stimulation to recover, allowing more persistent stimulation-associated epigenetic changes to be monitored. Epigenetic changes were evaluated through genome-wide profiling of DNAm using the Infinium MethylationEPIC microarray (Illumina, San Diego, CA). The DNAm data quality control (QC) of samples and CpGs is summarized in Additional file 1: Table S1, remaining 97 samples and 689,483 probes after the QC. The characteristics of samples passing the QC are summarized in Table 1. Briefly, participants from GALA II were Puerto Rican children and young adults and included similar proportions of healthy individuals, patients with mild asthma, and moderate-to-severe asthma.

**Table 1:** Characteristics of the airway cell donors

Variable	Discovery	Validation
	GALA II (* n* = 97)	Obese asthma study (*n* = 10)
Age (years)	13.9 (11.9–15.6)	28.0 (24.5–33.5)
Sex (female)	46 (47.4)	8 (80.0)
*Population*		
Hispanic	97 (100)	1 (10.0)
White	0 (0)	6 (60.0)
Asian	0 (0)	2 (20.0)
Asthma	67 (69.1)	5 (50.0)
*Asthma severity*		
Mild Asthma	31 (46.3)	NA
Moderate-to-severe Asthma	36 (53.7)	NA
BMI Categories*		
Underweight	4 (4.3)	0 (0.0)
Normal	51 (54.2)	4 (40.0)
Overweight	13 (13.8)	3 (30.0)
Obese	26 (27.7)	3 (30.0)
pre-FEV_1_ (liters)*	3.0 (2.4–3.5)	NA
pre-FVC (liters)*	3.4 (2.7–4)	NA
FEV_1_/FVC*	0.9 (0.9–0.9)	NA
BDR (%)*	9.2 (7.2–13.3)	NA
Total IgE*	455.1 (174.9–955.7)	NA
FeNO (ppb)	28.0 (13.0–50.2)	NA
Eosinophils (%)*	0.0 (0.0–0.1)	NA
*Medication use*^*†*^		
SABA	52 (77.6)	NA
Any controller medication	32 (47.8)	NA

Descriptives are represented by the median (interquartile range) for continuous variables and the count (proportion) for categorical variables.

*GALA II* Genes-environments & Admixture in Latino Americans Study; *NEC* Nasal epithelial cells; *BEC* bronchial epithelial cells; *BMI* body mass index; *FEV*_*1*_ forced expiratory volume in the first second; *FVC* forced vital capacity; *BDR* bronchodilator response; *IgE* immunoglobulin E; *FeNO* fractional exhaled nitric oxide; *SABA* short-acting beta-agonists. *NA* Not available

*In GALA II, BMI and lung function measurements were missing for 3 individuals, and IgE and eosinophils for 2 individuals.

^†^Only referred to asthma patients

### Genome-wide DNAm changes induced by albuterol treatment in nasal epithelia

**Fig. 2:**
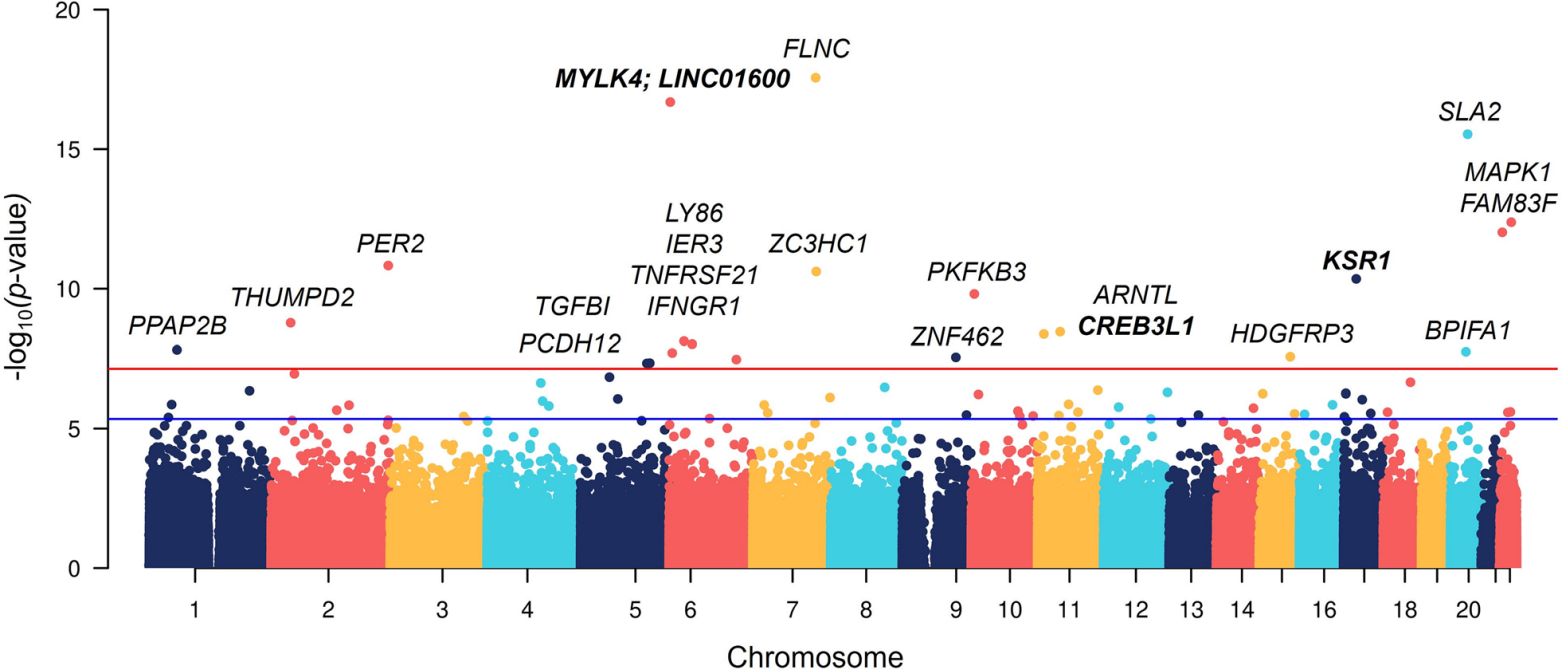
Manhattan plot of the EWAS of DNAm changes induced by albuterol treatment in nasal epithelial cells. The blue and red lines represent the false discovery rate (FDR) < 5% and the genome-wide (*p* < 9 × 10^–8^) significance thresholds, respectively. Gene annotation is represented for genome-wide significant CpGs, highlighting in boldface those CpGs that were validated

Albuterol-induced DNAm changes in nasal samples were assessed through a paired linear regression model. Analyses were corrected for cell-tissue heterogeneity, while all other potential confounders intrinsic to the sample were corrected by pairing (e.g., age, sex, asthma status, and ancestry). After bias and genomic inflation correction, the quantile–quantile (Q–Q) plots did not show evidence of genomic inflation (*λ* = 1.09; Additional file 1: Figure S1). A total of 66 CpG sites were associated with albuterol treatment with a false discovery rate (FDR) < 5%, and 22 CpGs surpassed the genome-wide significance threshold established for the EPIC array (*p* < 9 × 10^–8^) [18] (Fig. 2, Table 2, Additional file 1: Table S2). These included genes involved in inflammation (*TNFRSF21, IFNGR1, CSF3,* and *LTA4H*), interaction with the cytoskeleton (*FLNC, MYLK4, KSR1*, *IPP, SPTAN1, MACF1,* and *HSD17B12*), cell adhesion (*TGFBI, PCDH12, EPCAM, FAT4,* and *SPTAN1*), host defense against airway microbial infections (*BPIFA1*), and lipids metabolism and transportation (*PPAP2B, PLA2G6, ATP8B1, GLTPD2,* and *GRAMD1B*). Albuterol exerted predominantly a hypomethylation effect on CpG sites captured by the EPIC array across the genome (probability of hypomethylation: 0.76, 95% Confidence Interval [CI] 0.65–0.85, *p* value = 3.3 × 10^–5^) (Additional file 1: Figure S2).

**Table 2:** Summary results of genome-wide CpGs associated with albuterol exposure in nasal cells

CpG	Chr	Position*	Gene	Discovery			Validation		
				ΔDNAm (%)	logFC	*p*-value	logFC	*p*-value	FDR
cg10290200	7	128841241	*FLNC*	− 3.4	− 0.397	2.76 × 10^–18^	− 0.131	0.328	0.620
**cg00483640**	**6**	**2623249**	***MYLK4-LINC01600***	− **4.9**	− **0.358**	2.06 × 10^–17^	− **0.321**	**0.003**	**0.021**
cg15833511	20	36628395	*SLA2*	− 15.3	− 0.318	2.92 × 10^–16^	− − 0.169	0.102	0.348
cg24254891	22	39999311	*FAM83F*	− 15.6	− 0.290	4.16 × 10^–13^	− 0.015	0.906	0.963
cg12510044	22	21761184	*MAPK1*	− 9.1	− 0.277	9.56 × 10^–13^	− 0.151	0.152	0.368
cg02424494	2	238291487	*PER2*	− 2.3	− 0.219	1.49 × 10^–11^	− 0.202	0.031	0.133
cg03629778	7	130051485	*ZC3HC1*	− 10.1	− 0.203	2.43 × 10^–11^	0.039	0.701	0.851
**cg05673431**	**17**	**27544478**	***KSR1***	− **2.4**	− **0.175**	4.46 × 10^–11^	− **0.240**	**0.004**	**0.021**
cg11023970	10	6295886	*PFKFB3*	4.4	0.181	1.55 × 10^–10^	0.052	0.619	0.809
cg03956296	2	39724059	*THUMPD2*	− 17.7	− 0.475	1.66 × 10^–9^	NA	NA	NA
**cg23032799**	**11**	**46274563**	***CREB3L1***	− **6.5**	− **0.177**	3.48 × 10^–9^	− **0.264**	**1.53 × 10**^**–4**^	**0.003**
cg08161666	11	13013350	*ARNTL*	− 16.8	− 0.361	4.20 × 10^–9^	− 0.318	0.125	0.353
cg10310427	6	30749208	*IER3*	− 8.2	− 0.169	7.50 × 10^–9^	− 0.004	0.964	0.964
cg16769649	6	47358617	*TNFRSF21*	− 8.4	− 0.267	9.55 × 10^–9^	NA	NA	NA
cg26162522	1	56571443	*PPAP2B*	− 3.5	− 0.202	1.52 × 10^–8^	NA	NA	NA
cg16032470	20	33230393	*BPIFA1*	− 8.8	− 0.175	1.78 × 10^–8^	NA	NA	NA
cg03068616	6	6678265	*LY86*	− 12.6	− 0.271	1.97 × 10^–8^	− 0.174	0.202	0.429
cg16519100	15	83200514	*HDGFRP3*	23.5	0.271	2.66 × 10^–8^	− 0.121	0.478	0.734
cg22208174	9	106880861	*ZNF462*	− 7.6	− 0.239	2.81 × 10^–8^	NA	NA	NA
cg12845391	6	137224161	*IFNGR1*	− 4.4	− 0.129	3.38 × 10^–8^	− 0.043	0.518	0.734
cg12845808	5	141959039	*PCDH12*	− 7.3	− 0.109	4.54 × 10^–8^	− 0.012	0.866	0.963
cg03946667	5	136058672	*TGFBI*	− 5.2	− 0.147	4.63 × 10^–8^	− 0.052	0.505	0.734

*Chr* chromosome; *logFC* log_2_(fold-change); *SE* standard error; *FDR* false discovery rate

*Genomic positions are indicated in hg38/GRCh38 Genome Assembly.

Significant CpGs in the validation stage are in boldface

### Validation in independent samples and bronchial epithelia

Genome-wide significant CpGs were attempted for validation in an independent subset of nasal epithelial cells exposed under the same treatment conditions. The hypomethylation effect on three CpG sites annotated to the *CREB3L1* (cg23032799), *MYLK4-LINC01600* (cg00483640), and *KSR1* (cg05673431) genes was validated with an FDR < 5% (Table 2). In addition, these three CpGs from the nasal validation subset donors were cross-tissue evaluated in bronchial epithelial cells. The CpG cg23032799 (C*REB3L1*) showed a similar hypomethylation effect in bronchial epithelial cells exposed to repeated doses of albuterol (logFC = − 0.169, *p* = 0.030) (Additional file 1: Table S3).

### Evaluation of asthma-related conditions on albuterol-induced DNAm changes

We examined whether the albuterol-induced DNAm changes on CpGs could be partially conditioned by asthma status, previous use of asthma medications, or BDR during sample collection. First, a meta-analysis of stratified analysis based on asthma status (non-asthma, mild asthma, and moderate-to-severe asthma) did not report significant evidence of heterogeneity among groups (0.058 ≤ Cochran’s Q *p* value ≤ 0.916 and 0 ≤ *I*^2^ ≤ 64.94, Additional file 1: Table S4 and Figure S3). Similarly, stratified analyses based on the previous use of (i) albuterol or (ii) any controller medication, showed only one and two CpGs, respectively, out of the 22 evaluated with a significant heterogeneous effect according to previous medication use (Cochran’s Q *p* value < 0.05, Additional file 1: Tables S5, S6 and Figure S3).

Second, we applied a linear regression model to examine the effects of basal BDR on the albuterol-induced changes on DNAm in cell assays. Relative changes in DNAm induced by albuterol were estimated as follows: ΔDNAm = (DNAm_albuterol_−DNAm_control_)/DNAm_control_. Only asthma subjects had available BDR data. After adjusting for age, sex, ancestry, and tissue heterogeneity, DNAm changes on the CpG cg23032799 (*CREB3L1*) were associated with basal BDR. Specifically, lower basal BDR was associated with higher hypomethylation effect induced by albuterol (coefficient = 0.032, *p* = 0.004). However, this association did not remain significant after multiple comparisons adjustment (FDR = 0.09) (Additional file 1: Table S7).

### Identification of SNPs regulating changes on DNAm and gene expression

**Fig. 3:**
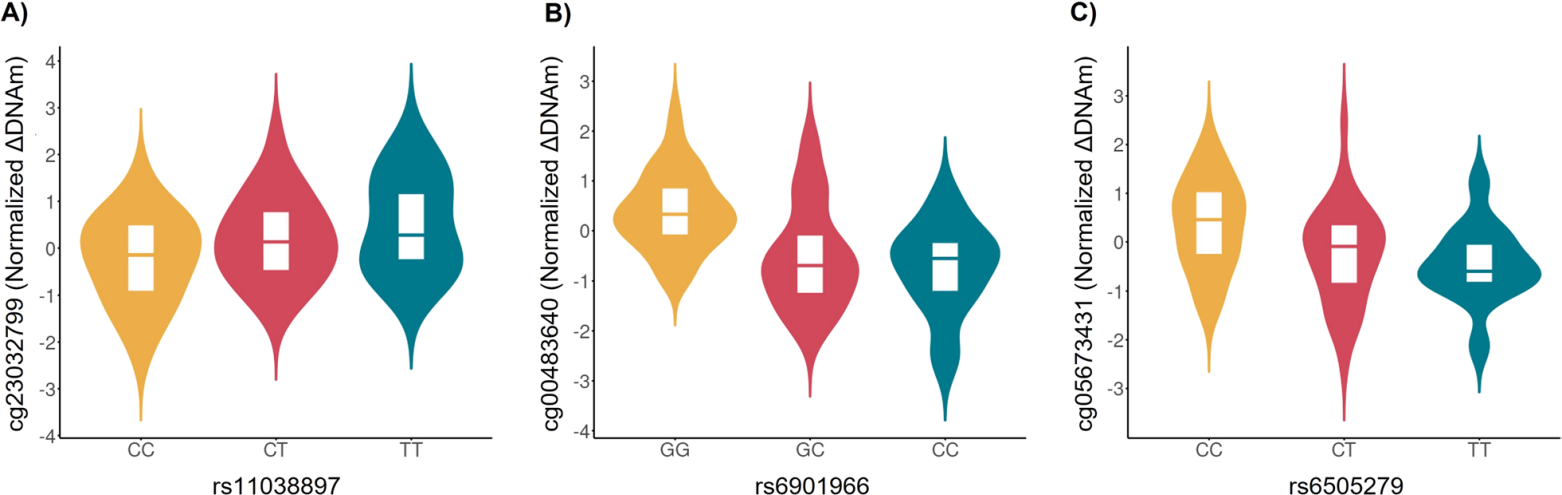
Violin plots of independent meQTLs in nasal samples identified for the genome-wide associated CpGs that were validated: **A** cg23032799 (*CREB3L1*), **B** cg00483640 (*MYLK4-LINC01600*), and **C** cg05673431 (*KSR1*). The normalized ΔDNAm values (*y*-axis) are plotted against the genotypes for each independent SNP

Whole-genome sequencing (WGS) data were available for individuals from the GALA II study [19]. A cis-methylation quantitative trait locus (meQTL) analysis was conducted to identify single nucleotide polymorphisms (SNPs) involved in the genetic susceptibility of albuterol-induced DNAm changes. Normalized ΔDNAm was included as the dependent variable and the genotypes of common SNPs as predictors. meQTL analyses were corrected for age, sex, ancestry, tissue heterogeneity, and asthma status. Only the three genome-wide associated CpGs validated in independent samples were associated with multiple meQTLs after multiple comparisons correction (FDR < 0.05, Table 3, Fig. 3, Additional file 1: Table S8).

**Table 3:** Summary results of independent meQTLs

CpG	Gene	rsID	Chr	Position*	Distance^†^ (bp)	A1	A2	MAF	Coefficient	SE	*p*-value	FDR
cg23032799	*CREB3L1*	rs11038897	11	46449277	174,714	T	C	0.281	0.491	0.134	4.24 × 10^–4^	0.047
cg00483640	*MYLK4-LINC01600*	rs6901966	6	2623299	50	C	G	0.245	− 0.757	0.128	6.83 × 10^–8^	1.20 × 10^–4^
cg05673431	*KSR1*	rs6505279	17	27454868	89,610	T	C	0.339	− 0.553	0.131	5.95 × 10^–5^	0.026

*rsID* reference SNP cluster ID; *Chr* chromosome; *A1* effect allele; *A2* non-effect allele; *MAF* minor allele frequency (effect allele); *SE* standard error; *FDR* false discovery rate

*Genomic positions are indicated in hg38/GRCh38 Genome Assembly.

^†^Distance between the CpG site and the SNP.

The functional consequences of independent meQTLs on gene expression in asthma-related tissues were evaluated through an in silico expression quantitative trait locus (eQTL) analysis. The SNP rs11038897, which regulates the DNAm changes on cg23032799 (*CREB3L1*), was found to regulate the gene expression of multiple genes in fibroblasts (*ATG13*, *MADD*, and *C11orf49*) and lung tissue (*ATG13*, *MDK*, and *C11orf49*). Additionally, the SNPs rs11038897 and rs6505279, which are meQTLs of the CpGs cg00483640 and cg05673431, respectively, regulate the expression of the genes where they are located in fibroblasts (*LINC01600*) and lung tissue (*KSR1*) (Additional file 1: Table S9).

### Functional consequences of DNAm changes on gene expression

Bulk RNA-seq data were generated in all paired samples from the discovery phase. An expression quantitative trait methylation (eQTM) analysis was carried out to test for the association between changes in DNAm and gene expression of nearby genes to each CpG. Relative changes in gene expression data were estimated as ΔRNA-seq = (RNA-seq_albuterol_−RNA-seq_control_)/RNA-seq_control_. The association between normalized ΔRNA-seq and ΔDNAm was tested through linear regression models adjusted for age, sex, asthma, ancestry, tissue heterogeneity, and batch effect from gene expression data. After multiple comparisons adjustment, we identified that hypomethylation at two CpGs was associated with increased gene expression patterns in the genes where they are located (Additional file 1: Table S10). Specifically, the CpGs cg10290200 and cg05673431 were associated with *FLNC* (coefficient: − 0.42, *p* = 0.001, FDR = 0.009) and *KSR1* (coefficient: -0.30, *p* = 0.010, FDR = 0.029) gene expression, respectively. The CpG cg05673431 was also associated with increased expression of *LGALS9* (coefficient: 0.27, *p* = 0.026, FDR = 0.039).

### Gene-set enrichment analysis

**Fig. 4:**
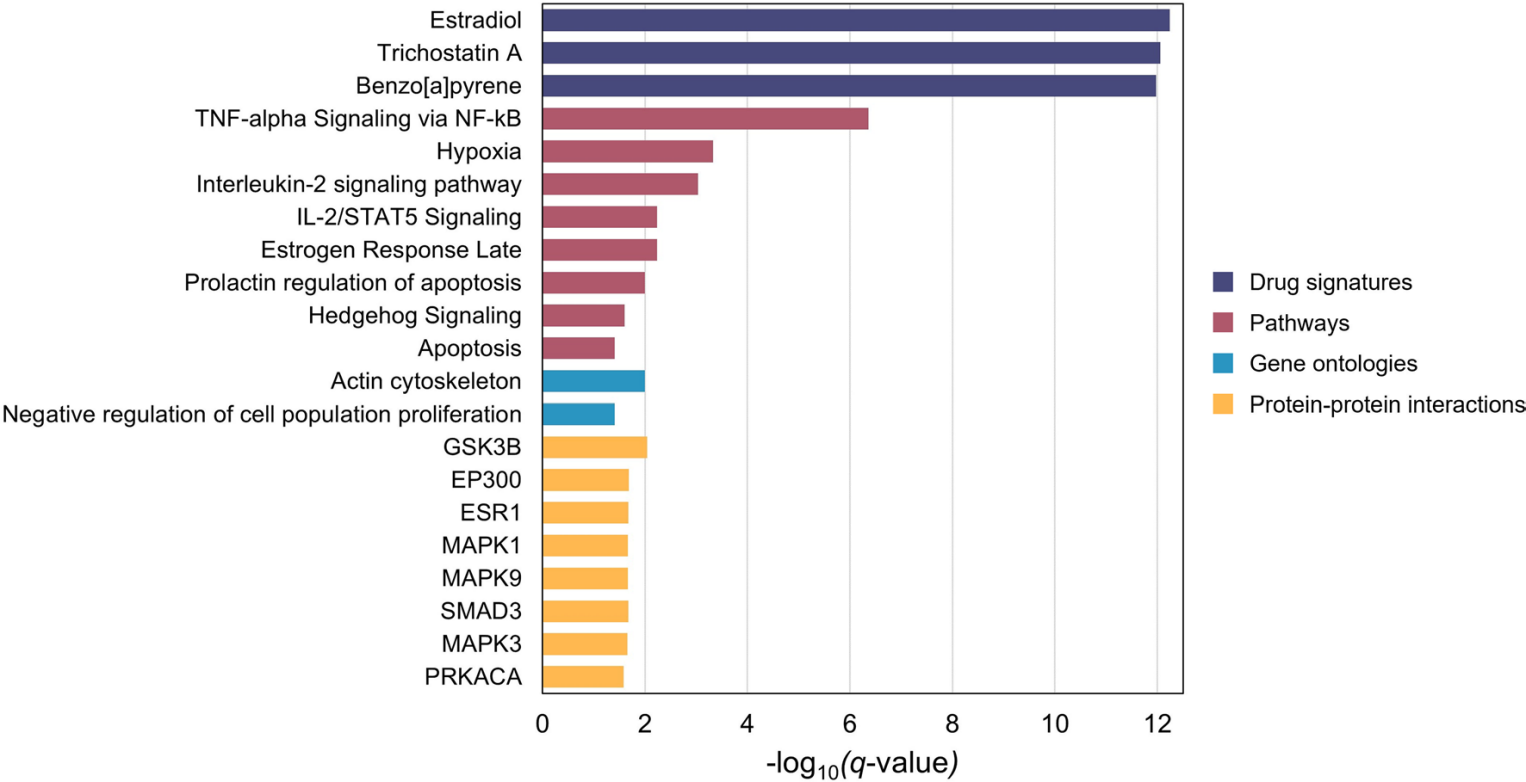
Bar plots of the significant results identified in the enrichment analyses using the Enrichr tool. The −log_10_(*q*-value) for each term is represented in the *x*-axis. Only the top-three significant terms of the drug signature enrichment analyses are reported.

We performed a gene-set enrichment analysis (GSEA) to identify biological pathways, molecular mechanisms, and drug signatures related to the epigenetic changes induced by albuterol and its functional consequences. Those CpGs that surpassed a *p* < 5 × 10^–4^ threshold in the main EWAS were selected to be annotated to genes, which were analyzed in the GSEA. As described in the Methods section, we only retained the results that remained significant after multiple comparison corrections (FDR < 0.05) and showed to be robust to varying the input *p* value thresholds for selecting the CpGs. We observed enrichment in multiple biological pathways and gene ontologies potentially related to asthma and the mechanism of action of albuterol. These included the tumor necrosis factor-alpha (TNF-α) signaling pathway mediated by the nuclear factor kappa B (NF-kB) transcription factor, interleukin-2 (IL-2) signaling pathway, regulation of cell proliferation, and components of the actin cytoskeleton. Additionally, we reported that genes affected by albuterol through DNAm changes are more likely to be regulated by numerous compounds and drugs than chance, including estradiol and trichostatin A (TSA) (Fig. 4, Additional file 1: Table S11).

### Regional DNAm changes induced by albuterol

We examined whether repeated stimulations with albuterol induce changes in DNAm on genomic regions. Differentially methylated regions (DMRs) were estimated using two independent software to minimize false positive results. Regional DNAm changes induced by albuterol were identified in nine DMRs (adjusted *p* value < 0.05, Table 4, Additional file 1: Tables S12, S13). The top hit was a genomic region of 103 bp annotated to the *GLTPD2* gene (adjusted *p* value = 3.75 × 10^–12^). We also identified DMRs annotated to *CREB3L1* (adjusted *p* value = 9.81 × 10^–10^), *MYLK4* (adjusted *p* value = 2.04 × 10^–7^), and *KSR1* (adjusted *p* value = 0.007).

**Table 4:** DMRs associated with albuterol treatment in nasal samples

Gene	Chr	Start*	End*	Width	No of CpGs	Adjusted *p* value
*GLTPD2*	17	4788081	4788183	103	3	3.75 × 10^–12^
*CREB3L1*	11	46274469	46274643	175	4	9.81 × 10^–10^
*MYLK4*	6	2623249	2623545	297	2	2.04 × 10^–7^
*CABYR*	18	23992402	23992785	384	5	7.86 × 10^–5^
*NDRG1*	8	133298310	133298476	167	3	1.33 × 10^–4^
*PER2*	2	238300705	238300990	286	3	5.98 × 10^–4^
*FOXQ1*	6	1228715	1228752	38	2	0.001
*KSR1*	17	27544478	27544765	288	2	0.007
*DAZL*	3	16605476	16605619	144	4	0.013

*Genomic positions are indicated in hg38/GRCh38 Genome Assembly

*DMR* differentially methylated region; *Chr* chromosome

## Discussion

In this study, we identified for the first time epigenomic changes induced by albuterol in mucociliary airway epithelia. Repeated stimulations with albuterol induced an overall genome-wide hypomethylation on CpGs captured by the EPIC array in nasal epithelia. Among 22 genome-wide significant CpGs, the effects on three CpGs annotated to *CREB3L1*, *MYLK4-LINC01600*, and *KSR1* genes were validated in independent nasal samples. Albuterol induced regional DNAm changes in at least nine DMRs, including *CREB3L1*, *MYLK4-LINC01600*, and *KSR1*. The hypomethylation effect of the CpG near *CREB3L1* was cross-tissue validated in bronchial epithelia and suggestively associated with BDR. Specifically, we observed that the hypomethylation effect on *CREB3L1* was inversely associated with BDR, suggesting that albuterol might have further functional consequences in the gene regulatory landscape in poor responders to bronchodilators. We also reported meQTLs associated with the genetic susceptibility to these albuterol-induced DNAm changes, which regulate gene expression of nearby genes in lungs and/or fibroblasts. Additionally, hypomethylation on the CpGs cg10290200 and cg05673431 was associated with higher expression of *FLNC* and *KSR1*, respectively. Moreover, epigenetic loci modified by albuterol were located in genes enriched in asthma-associated processes (i.e., TNF-α, NF-kB, and IL-2 signaling pathways) and likely regulated by a potential asthma drug (i.e., TSA).

Albuterol triggers a rapid-onset bronchodilator effect in the airway smooth muscle and regulates airway inflammation by diverse pathways that have not been completely uncovered. The main known mechanism of action is mediated by its agonism on the β_2_-adrenergic receptor (ADRB2) [20]. Briefly, the activation of ADRB2 initiates a cascade of intracellular signals characterized by increasing cyclic adenosine monophosphate (cAMP) intracellular levels and resulting in the inhibition of intracellular Ca^2+^ via protein kinase A. The bronchodilator effect is a consequence of the inhibition of the Ca^2+^-dependent myosin light chain phosphorylation [20]. The main epigenetic markers reported in this study are annotated to genes potentially involved in this pathway. Our findings are relevant by providing new insights into potential mechanisms and epigenomic cell responses induced by albuterol that have not been described yet.

First, we reported albuterol-induced DNAm changes on *CREB3L1* in nasal and bronchial epithelial cells, which are associated with genetic variation and the BDR of the donors. *CREB3L1* encodes the cAMP response element binding protein 3-like 1, a member of the CREB/ATF transcription factor family. CREB3L1 is activated in response to increased cAMP levels and is involved in stress response, regulation of cell secretory capacity, extracellular matrix production, cell migration, and virus infection response [21–24]. Interestingly, we recently found *CREB3L1* to be a transcription factor expressed in human mucus secretory cells [25]. Our finding of albuterol-induced downregulation in *CREB3L1* methylation locus, suggests that airway secretory cell differentiation could be modulated by albuterol usage. DNAm on *CREB3L1* participates in the toxic effects of inhaled silica nanoparticles on bronchial epithelial cells [26]. Although genetic variants in the intergenic region of *PHF21A*-*CREB3L1* have been associated with post-bronchodilator lung function measurements [27], the potential role of DNAm has not been described. Nonetheless, the family-related CREB1 protein has been widely studied in the context of asthma. CREB1 participates in promoting epigenetic changes to switch on pro-inflammatory genes and is involved in persistent bronchial inflammation in asthma and the anti-inflammatory effect of inhaled corticosteroids [28]. Additionally, it participates in the regulation of *ADRB2* expression and downregulates lung receptors after chronic exposure to β_2_-agonists [29]. CREB1 is one of the proteins involved in airway smooth muscle contraction mediated by cAMP and diacylglycerol kinase (DGK) [30]. Although the effects in vivo are uncertain, in vitro models have shown that β_2_-agonists increase CREB1 binding to the DNA in murine lung cells, while corticosteroids reduce it [31, 32]. Similarly, *CREB3L1* expression is diminished in the rat hypothalamus after dexamethasone exposure, while increased levels of cAMP enhance its expression [33].

Second, we reported DNAm changes, influenced by genetic variation, on epigenetic markers annotated to *MYLK4* and *KSR1*. Additionally, we also identified that albuterol induced hypomethylation on two CpGs associated with increased gene expression of *FLNC* and *KSR1*. *MYLK4* encodes a member of the myosin light chain kinase (MYLK) family. MYLKs are involved in the phosphorylation of the myosin light chain and promote the contraction of smooth muscle cells leading to bronchoconstriction [34]. Although studies on *MYLK4* are limited, MYLKs are known to participate in cell migration, invasion, and proliferation [34]. Genetic variation in *MYLK1*, the best-studied gene in the *MYLK* family, is associated with asthma risk and exacerbations and is a common genetic factor in diseases involving smooth muscle contraction and inflammation [35, 36]. The kinase activity of MYLK is activated by calmodulin when is bound to Ca^2+^ [37]. The kinase suppressor of ras 1 (KSR1) is a scaffold protein required for the association between Ca^2+^ and calmodulin, with a known regulatory effect on the mitogen-activated protein kinase (MAPK) cascade [38]. On the other hand, the filamin C (FLNC) is a protein that participates in the interaction between actin filaments and binding partners in muscle cells [39]. Although its role in the ADRB2 pathway has not been described, FLNC interacts with β-arrestin-2 [39], which is one of the main mediators involved in the side effects of bronchodilators via ADRB2 [20].

Third, enrichment analyses revealed that epigenomic modifications of albuterol could participate in IL-2, TNF-α, and NF-κB pathways, and that gene expression levels of affected genetic loci were more likely to be regulated by TSA and estradiol than expected by chance. IL-2 is a pro-inflammatory cytokine involved in the development of Treg cells and associated with eosinophil proliferation, airway inflammation and hyperreactivity, and impaired lung function in asthma patients [40–42]. IL-2 is involved in airway smooth muscle contraction by regulating the airway sensitization to leukotrienes [42], and IL-2-based therapies induce severe bronchoconstriction that can be reversible with albuterol under certain conditions [43]. Additionally, the effects of albuterol on airway inflammation might be mediated by IL-2 through the inhibition of TNF-α and NF-κB, and the activation of type 2 inflammation via STAT5 [44, 45]. NF-κB is a pro-inflammatory transcription factor widely involved in the pathogenesis of asthma [44] whose activation is inhibited by β_2_-agonists [46]. TNF-α is a cytokine with a regulatory role in inflammatory responses and the development of allergic diseases, particularly asthma [40]. TNF-α can activate NF-κB-mediated pathways and its expression is increased in patients with severe and steroid-refractory asthma, being considered a potential therapeutic target [47, 48]. Similarly, albuterol inhibits TNF-α expression, a mechanism involved in the agonism of the anti-inflammatory effect of corticosteroids and theophylline [49, 50]. Finally, TSA is an inhibitor of histone deacetylases that regulates inflammatory genes via NF-κB and has demonstrated in vivo anti-inflammatory properties in murine asthma models [51]. It has also been shown to reduce airway constriction by decreasing Ca^2+^ mobilization [52]. Additionally, previous studies have reinforced its potential therapeutic interest in asthma exacerbations by regulating genes implicated in ICS response and microbiome composition [53, 54]. On the other hand, estradiol is one of the main sex hormones in females, which has been suggested to be implicated in the sex differences in asthma throughout the lifespan [55]. Although this finding may suggest a differential in vitro effect of albuterol between cells derived from females and males, except for probe cg15833511, we did not observe evidence of significant heterogeneity in stratified analyses based on biological sex (Additional file 1: Table S14).

This study has several strengths. First, our experimental design allowed us to identify the causality between albuterol exposure and airway methylome alterations. The sample size in the discovery stage provided an 80% statistical power to identify modest DNAm changes (≥ 10%) with a *p *value < 1 × 10^–6^ (which approximately corresponds to a genome-wide significance at an FDR < 5%) [56]. Second, we applied a discovery, validation, and cross-tissue evaluation design that supported the robustness of our associations and their potential effects in different asthma-relevant tissues. Third, potential spurious results were minimized by correcting for bias and genomic inflation using a Bayesian method designed for EWAS and controlling for potential cell heterogeneity and unknown confounders. Fourth, we integrated epigenomic, transcriptomic, and WGS data mainly focusing on a diverse and underrepresented population (i.e., Puerto Ricans). Nevertheless, some limitations must be acknowledged. First, the sample size in the validation stage is limited, restricting the statistical power for the validation of markers identified in the discovery phase. Since independent donors were balanced for diverse relevant characteristics (i.e., age, sex, asthma status, body mass index, and ethnicity), we were unable to assess phenotype-specific effects with evidence of validation. Second, although it is recognized that the nasal airway reflects the lower airways, the effects on bronchial epithelial cells remained partially understudied in our study due to a limited sample size. Third, despite examining the potential influence of the prescription of asthma medications, the frequency of use and medication adherence were not available. Fourth, our study design did not allow us to identify potential clinical implications of epigenetic changes on treatment response, adverse reactions, and/or drug tolerance. Fifth, identified epigenetic markers in vitro may not reflect in vivo effects of albuterol when administered at a therapeutical dosage in asthma patients. Human organoids and in vivo animal models should allow further investigation of potential effects taking place in a therapeutic context. Sixth, the effect of albuterol on sex chromosomes DNAm remained unaddressed in this study.

## Conclusions

We characterized for the first time the epigenomic response induced by albuterol treatment in airway epithelia. DNAm in 22 genome-wide significant CpGs and nine genomic regions were modified by albuterol in nasal epithelia from Puerto Rican children with different asthma statuses. Affected genes were enriched in asthma-relevant processes, including IL-2, TNF-α, and NF-κB signaling pathways, and were more likely to be regulated by Trichostatin A than chance. We observed evidence of genetic susceptibility to albuterol-induced changes and functional consequences on *FLNC* and *KSR1* expression. Our findings provide novel insights into the potential role of *CREB3L1*, *MYLK4*, *KSR1*, and *FLNC* on epigenetic-mediated biological effects induced by albuterol.

## Methods

### Human subjects information

Basal airway epithelial cultures and then subsequently derived ALI cultures, used for the 100 donors' repeated-use albuterol stimulation model, were derived from nasal airway epithelial brushings collected from subjects recruited as part of the Genes-environments and Admixture in Latino Americans II (GALA II) childhood asthma study, which was approved by local institutional review boards (UCSF, IRB number 10–00889, Reference number 153543, NJH HS-2627). All subjects and their parents provided written informed assent and written informed consent, respectively [57]. Paired nasal and bronchial airway brushings used in generating the ALI albuterol validation study cultures were derived from subjects recruited as part of the Obese Asthma: Unveiling Metabolic and Behavioral Pathways study at CU-Anschutz and approved by local institutional review boards (University of Colorado, IRB Number 19-0510, 21-3959, and 16-2522, NJH HS-3110).

### Albuterol treatment of mucociliary airway epithelial cultures

To model the in vivo airway epithelium, we generated ALI mucociliary epithelial cultures. For primary human nasal and bronchial epithelial samples, basal cells were cultured for expansion using a modified Schlegel method as previously described [58, 59]. Primary basal cells were seeded onto 6.5 mm, 0.4 μm pore transmembrane inserts (4 × 10^4^ cells/insert) in PneumaCult Expansion Plus medium (StemCell Technologies) supplemented with Y-27632. Cultures were air-lifted upon confluence and basolateral media was then switched to PneumaCult ALI (PC-ALI; StemCell Technologies) media to stimulate mucociliary differentiation over the next 24 days.

Albuterol stimulations were completed on cultures from nasal samples from the GALA II study (*n* = 100 total; 30 and healthy controls, 33 with mild asthma, and 37 with moderate-to-severe asthma) and paired nasal and bronchial samples from the Obese Asthma study (*n* = 10 total; five healthy controls and five asthma patients). Exposures were performed starting at ALI D24 for epithelial cultures and stimulations were performed twice daily, once in the morning, and once in the evening for five consecutive days. For epithelial cultures, each morning fresh basolateral media was added to all ALI inserts, apical chambers were washed with warm PBS, and 20μl of ALI media supplemented with MeOH as vehicle control or ALI media supplemented with 100μM albuterol was added to the apical chamber of each ALI culture. For evening stimulations, apical chambers were washed once with warm PBS, and stimulations were performed as described above. PBS washes were performed prior to re-stimulations to prevent to accumulation of albuterol over time. Following 5 days of albuterol stimulations, ALI received fresh culture media without vehicle or albuterol and were allowed to rest for 48 h to allow for interrogation of epigenetic changes resulting from albuterol exposure. Samples were lysed using Zymo DNA/RNA Lysis Buffer (Zymo Research, Irvine, USA) supplemented with 40 mM DTT, and triplicates were pooled for DNA and RNA isolations as per manufacturer instructions.

### Genome-wide DNAm profiling and quality control

Whole-genome DNAm data from 856,553 CpGs was generated using the Infinium MethylationEPIC microarray (Illumina) following the manufacturer's recommendations. QC of DNAm data was performed using the *ENmix* and *ewastools* R packages, as previously described [14, 60–62]. Briefly, samples with (1) a high proportion of bad-quality methylation data (> 5% of CpGs), (2) discordance between the reported sex and the epigenetic predicted sex, (3) a high missingness rate (> 5% of CpGs), and (4) potential cross-sample contaminated (based on the genotype distribution of control probes) were removed. Considering the paired approach of this study, samples were also removed if their paired sample was discarded. After QC, a total of 97 nasal cells were retained for the discovery stage, and all 10 nasal and bronchial epithelial cells were kept for the validation analyses. A QC of CpG sites was also conducted using the *ENmix* package [62]. Briefly, we corrected bisulfite intensities for background noise, dye and probe-type biases (oob and RELIC methods), and inter-array heterogeneity (quantile normalization) [63, 64]. Then, we excluded probes (1) with a high proportion of bad-quality methylation data (> 5% of samples), (2) located in sex chromosomes, (3) that non-specifically bind to a genomic position (cross-reactive) [65, 66], (4) with a multimodal distribution, and (5) potentially polymorphic probes. Probes with a multimodal distribution of beta values, which could capture other artifacts different than DNAm, were identified using the *nmode* function from *ENmix* [62]. Polymorphic probes were defined as probes containing a common SNP with a minor allele frequency (MAF) ≥ 5% at the CpG site (either at cytosine or guanine nucleotides) and, in the case of Type I probes, also those with a common SNP at the position where single base extension takes place during the methylation assay. We used available WGS data from GALA II to identify SNPs and estimate MAFs specifically in the analyzed individuals. In samples from independent donors (validation stage), we used the data from the Illumina manifest file v1.0 B4 [67]. DNAm was computed as beta values and transformed into M-values for better statistical performance. QC was conducted separately in the discovery and validation sets. Probes with a multimodal distribution were independently assessed for each cell type.

We used the ReFACTor algorithm as a free-reference method to control for unknown variation, mainly driven by cell heterogeneity [68]. The appropriate number of ReFACTor components to include in subsequent analyses (*n* = 3) was estimated for each dataset based on scree plots following the software developers’ recommendations. ReFACTor components were estimated with adjustment for cell condition (albuterol *vs.* control) and controlling for pairing. The *sva* R package was used to estimate surrogate variables for batch effect correction, but no significant variables in our dataset were detected [69].

### Epigenome-wide association study

The discovery phase included the largest dataset consisting of 97 paired nasal epithelial cells from Puerto Rican children included in the GALA II study. We assessed the effect of albuterol treatment on whole-genome DNAm through paired robust linear regression models using the *limma* R package [70]. Models were corrected for ReFACTor principal components, while all other potential confounders intrinsic to the sample were corrected by pairing (e.g., age, sex, asthma status, ancestry). Considering that methylation levels were log-transformed into M-values, the log_2_(Fold-change) or logFC parameter from *limma* corresponds to the difference in the average M-value between control and treated samples. Negative and positive logFC values indicate the hypomethylation and hypermethylation effects of albuterol, respectively, and their values are correlated with the magnitude change in methylation values. We used the *bacon* R package to correct the effect sizes, standard errors, and *p* values controlling for bias and genomic inflation in epigenomic studies [71]. After correction, genomic inflation was further inspected by examining the Q–Q plots and the genomic inflation factor (*λ*). An FDR < 5% was used to declare significance and minimize false positive results, and the genome-wide significant threshold was established in *p* < 9 × 10^–8^, as previously estimated for the EPIC array [18]. Those significant CpGs with changes on DNAm (expressed as beta-values) < 2% were flagged as being potentially related to technical variations [72]. CpGs were annotated based on the Illumina manifest file v1.0 B4 and using the Genomic Regions Enrichment of Annotations Tool (GREAT) v4.0.4 [67, 73]. A one-sided binomial test was applied to assess whether albuterol showed predominantly a hypomethylation or hypermethylation effect. Genome-wide significant CpGs were followed-up in all subsequent analyses.

### Validation in an independent subset of nasal and bronchial epithelial cells

DNAm changes induced by albuterol were attempted for validation in a subset of independent nasal epithelial cells from 10 donors treated under the same albuterol exposure conditions. An FDR < 5% was used to declare a significant association in this validation stage. Genome-wide CpGs that were validated were followed up in a cross-tissue evaluation analysis using bronchial epithelial cells. In these samples, we tested for the association of changes in DNAm and albuterol exposure following the same procedure as in nasal samples. A nominal *p* < 0.05 was used to declare a significant association.

### Evaluation of asthma-related conditions on albuterol-induced DNAm changes

We examined whether albuterol-induced DNAm changes were conditioned by asthma status, biological sex, previous use of asthma medications, or BDR status during sample collection. Asthma status was classified according to the diagnosis and severity of the disease in non-asthma subjects, mild asthma patients, and moderate-to-severe asthma patients. Asthma severity was derived from the control of asthma symptoms (assessed using the Asthma Control Test and the Asthma Control Questionnaire) and the therapeutic step of each patient according to the Expert Panel Report-3 guidelines for diagnosis and management of asthma [74]. Mild intermittent and mild persistent asthma categories were classified as mild asthma, while moderate persistent and severe persistent asthma categories were grouped into moderate-to-severe asthma. Regarding asthma medications, we evaluated having a prescription of (1) short-acting beta-agonists or (2) any controller medication (i.e., inhaled corticosteroids, long-acting beta-agonists, combo medication, leukotriene receptor antagonists, oral corticosteroids, and/or theophylline). BDR was clinically characterized in asthma patients during sample collection as the maximum relative improvement in lung function (i.e., forced expiratory volume in the first second or FEV_1_) after albuterol inhalation [8].

We conducted stratified analyses according to asthma status, use of asthma medication, and biological sex and meta-analyzed them using METASOFT [75]. The heterogeneity among groups was examined using Cochran’s Q *p* value and *I*^2^. A Cochran’s Q *p* value < 0.05 or > 0.05 indicates the presence or absence of heterogeneity, respectively. The *I*^2^, which ranges between 0 and 100%, measures the proportion of variance as a consequence of the observed heterogeneity. The higher the *I*^2^ value, the greater the presence of heterogeneity.

On the other hand, linear regression models were applied to examine the effects of basal BDR on albuterol-induced DNAm changes in the cell assays. The relative change in DNAm induced by albuterol was estimated for each subject and CpG site as follows: ΔDNAm = (DNAm_albuterol_-DNAm_control_)/DNAm_control_. The ΔDNAm was normalized by applying an inverse normal transformation to ensure a normal distribution. Considering the inter-individual comparison of this approach, models were corrected for age, sex, ancestry (the first three principal components), and cell heterogeneity (the first three ReFACTor components). ReFACTor components were re-estimated using the DNAm values from control samples adjusting for BDR, age, sex, and ancestry. Multiple comparisons were adjusted using an FDR < 0.05.

### Methylation quantitative trait loci analyses

WGS data were generated for Puerto Ricans from GALA II within the Trans-Omics for Precision Medicine (TOPMed) Consortia at the New York Genome Center and Northwest Genomics Center. Briefly, paired-end reads (150 bp × 2) were generated on the HiSeq XTM Ten platform (Illumina) reaching a minimum mean genome coverage of 30x. All technical details are described on the TOPMed website [19]. The genetic susceptibility of albuterol-induced DNAm changes was investigated through a meQTL analysis. A total of 96 individuals with available epigenomic and WGS data were included in the analysis. For each CpG site, all SNPs located within a window of 500 kb (± 250 kb upstream and downstream) with a MAF ≥ 5% were evaluated in the cis-meQTL analysis. The association between normalized ΔDNAm and genotypes of these SNPs was tested through linear regression models using fastQTL that were adjusted for age, sex, ancestry, asthma status, and tissue heterogeneity [76]. An FDR < 0.05 at the probe level was used to correct for multiple comparisons considering that each CpG maps to multiple SNPs. Independent meQTL signals were identified by conditional regression models conditioned on the most significant meQTL using PLINK 1.9, as previously described [14]. Briefly, meQTL analyses were repeated adding the corresponding top-hit meQTL for each CpG as a covariate. All non-significant (*p* > 0.05) meQTLs were filtered out since they are dependent on the top-hit signal. If any meQTL remained significant, the new top-hit was considered as another independent meQTL and the process was repeated until no significant meQTLs were identified.

### In silico expression quantitative trait loci analyses

The effects on gene expression of independent meQTL were inspected through in silico eQTL analyses using the public database Genotype-Tissue Expression (GTEx) portal. We examined whether these SNPs associate with the expression levels of nearby genes in different tissues related to the respiratory system. Summary statistics were extracted from the GTEx portal.

### Expression quantitative trait methylation analyses

RNA normalization and library construction were performed using the KAPA mRNA Hyper Prep Kit (Roche, Basel, Switzerland), using the Beckman Coulter FXp automation system. Briefly, 200 ng of RNA was used as input, and Illumina Dual-Index adaptors from Integrated DNA Technologies were used to barcode libraries using 12 cycles of amplification. Paired-end sequences from pooled libraries were obtained using the NovaSeq 6000 platform (Illumina). Regarding the pre-processing of bulk RNA-seq data, raw reads were trimmed with bbduk.sh from the BBMap package v38.79 with parameters “trimq = 10 qtrim = r minlength = 70 ref = adapters ktrim = r k = 23 mink = 11 hdist = 1 tpe tbo” [77]. Transcript counts were quantified with Salmon v1.5.2 with parameters “—seqBias —gcBias” [78] using human genome reference Gencode [https://doi.org/10.1093/nar/gkaa1087] release 38 (GRCh38.p13). The Salmon output was imported into R and transcript-level counts aggregated to gene-level with *tximport* [79]. A total of 198 RNA-seq samples were used for analysis. One donor was removed due to the control and treatment labels being swapped. Genes were included if they were protein-coding and had an inferred count of at least 6 in at least 10% of the samples. Expression counts were transformed with the variance stabilizing transformation (VST) using *DESeq2* v1.34.0 [80].

The association between albuterol-induced DNAm and gene expression changes was examined in a cis-eQTM analysis. Individuals with available DNAm and RNA-seq data (*n* = 95 paired samples) were included in the analysis. Changes in gene expression (ΔRNA-seq) were estimated and normalized following the same procedures as with DNAm data. We estimated surrogate variables of unknown sources of variation to correct for batch effect in RNA-seq data using the *sva* R package [69]. The significant number of surrogate variables was estimated using the *svaseq* function while adjusting for age, sex, ancestry, and tissue heterogeneity. To minimize overfitting in the eQTM analysis, only those surrogate variables not significantly correlated with any ReFACTor principal component were retained for the analyses (*p* > 0.05) (Additional file 1: Figure S4). Similar to ReFACTOr, surrogate variables were estimated using the gene expression data from the control samples. Then, we tested for the association between the normalized ΔDNAm of each CpG site and the normalized ΔRNA-seq of all genes whose transcription start site is located ± 250 kb upstream and downstream of each CpGs using *MatrixEQTL* [81]. Models were corrected for age, sex, asthma, ancestry, tissue heterogeneity, and RNA-seq batch effect. An FDR < 0.05 at the probe level was used to correct for multiple comparisons considering that each CpG maps to multiple genes.

### Enrichment analyses

A GSEA was conducted to investigate whether the associated CpGs from the discovery EWAS were annotated to genes significantly involved in biological pathways, gene ontologies, traits, and drug signatures. Based on the EWAS summary statistics, a *p* < 5 × 10^–4^ threshold was used to select CpGs to include in the enrichment analyses. The GSEA was performed using the Enrichr database and an FDR < 0.05 was used to correct for multiple comparisons [82]. The GSEA was re-assessed by varying the *p* value thresholds for CpGs selection (i.e., *p* < 5 × 10^–5^ and *p* < 5 × 10^–3^). Only the terms that remained significant (*p* < 0.05) using these two alternative thresholds were retained to ensure the robustness of the findings. Analyses were carried out in January 2023, with the available version of the following gene-set libraries in Enrichr: BioPlanet 2019, Molecular Signatures Database (MSigDB), Gene Ontologies (GO) Biological Process 2019, GO Cellular Components 2019, Protein–Protein Interactions (PPI) Hub dataset, Transcription Factor PPIs, PheWeb 2019, and Drug Signature Database (DSigDB).

### Differentially methylated regions

Regional DNAm analyses were conducted to identify DMRs associated with albuterol-induced changes in DNAm. We identified DMRs using two independent software: comb-p and *DMRcate* [83, 84]. DMRs were started at CpGs individually associated with *p* < 0.05 (comb-p) or FDR < 0.05 (*DMRcate*) and significant CpGs subsequently located in sliced windows of 1000 bp were included in the same region. All regions were restricted to contain at least two CpGs. In *DMRcate* the scaling factor for bandwidth was used as recommended by the developers (*C* = 2). Multiple comparisons were corrected intrinsically by each software using a Šidák-corrected *p* < 0.05 in comb-p and FDR < 0.05 in *DMRcate*. To minimize false positive results, only DMRs identified using the two software were retained. DMRs were annotated to the single nearest gene using GREAT v4.0.4 [73].

### Supplementary Information

**Additional file 1**. Supplementary material (Supplementary Figures S1-S4 and Supplementary Tables S1-S14).

## Data Availability

All data necessary to evaluate the conclusions of this manuscript are reported in the main text and/or the supplementary information. Raw methylation data of samples included in the discovery phase (GALA II) are publicly available in the Gene Expression Omnibus (GEO) database under the accession number (GSE240155). TOPMed whole-genome sequencing data from GALA II are available in the database of Genotypes and Phenotypes (dbGaP) under accession number phs000920.v1.p1. The summary statistics of the epigenome-wide association study and the full report of the gene-set enrichment analysis are available in the Zenodo repository (https://doi.org/10.5281/zenodo.7261818).

## Abbreviations

95% CI: 95% Confidence interval
ADRB2: β_2_-Adrenergic receptor
ALI: Air–liquid interface
BDR: Bronchodilator drug response
cAMP: Cyclic adenosine monophosphate
CpG: Cytosine-phosphate-guanine dinucleotide
DGK: Diacylglycerol kinase
DMR: Differentially methylated region
DNAm: DNA methylation
eQTL: Expression quantitative trait locus
eQTM: Expression quantitative trait methylation
EWAS: Epigenome-wide association study
FDR: False discovery rate
GALA II: Genes-environments and Admixture in Latino Americans
GSEA: Gene-set enrichment analysis
GTEx: Genotype-tissue expression portal
GWAS: Genome-wide association study
IL-2: Interleukin-2
log_2_FC: Log_2_(fold-change)
MAPK: Mitogen-activated protein kinase
meQTL: Methylation quantitative trait locus
MYLK: Myosin light chain kinase
NF-κB: Nuclear factor kappa B
QC: Quality control
Q–Q plot: Quantile–quantile plot
SNP: Single nucleotide polymorphism
TNF-α: Tumor necrosis factor-alpha
TSA: Trichostatin A
WGS: Whole-genome sequencing
