# Supplementary material for: Epigenomic response to albuterol treatment in asthma-relevant airway epithelial cells
Source: Clin Epigenetics. 2023 Oct 3;15:156. doi: 10.1186/s13148-023-01571-0 (PMC10546710; doi:10.1186/s13148-023-01571-0)
Supplement: Supplementary file 1 — Additional file 1. Supplementary material (Supplementary Figures S1-S4 and Supplementary Tables S1-S14). [file 13148_2023_1571_MOESM1_ESM.docx]

***Supplementary Material***

**Epigenomic Response to Albuterol Treatment in Asthma-Relevant Airway Epithelial Cells**

***Running title:*** *Epigenomic Response to Albuterol*

Javier Perez-Garcia, PharmD^*^, Maria Pino-Yanes, PhD^*^, Elizabeth G. Plender, BSc, Jamie L. Everman, PhD, Celeste Eng, BSc, Nathan D. Jackson, PhD, Camille M. Moore, PhD, Kenneth B. Beckman, PhD, Vivian Medina, RN, Sunita Sharma, MD, Daniel Efrain Winnica, PhD, Fernando Holguin, MD, José Rodríguez Santana, MD, Jesús Villar, MD, PhD, Elad Ziv, MD, Max Seibold, PhD^†^, Esteban G. Burchard, MD, MPH^†^

*^†^Equal contribution as senior authors*

****Corresponding authors***

*Javier Perez-Garcia, PharmD and Maria Pino-Yanes, PhD.*

*Genomics and Health Group, Department of Biochemistry, Microbiology, Cell Biology, and Genetics, Universidad de La Laguna. Apartado 456, La Laguna, 38200 Tenerife, Canary Islands, Spain.*

*Tel: +34 922316502–6343*

*Email:* [*jpegarci@ull.edu.es*](mailto:jpegarci@ull.edu.es)*;* [*mdelpino@ull.edu.es*](mailto:mdelpino@ull.edu.es)

This Supplementary Material file contains the following items:

| **TABLE OF CONTENTS** | **PAGES** |
| --- | --- |
| **Supplementary Figures** |  |
| Figure S1. QQ plot of the discovery phase. | 3 |
| Figure S2. Volcano plot of the discovery phase. | 4 |
| Figure S3. Forest plot of asthma-status stratified analyses. | 5 |
| Figure S4. Correlation plot of ReFACTor components and surrogate variables | 6 |
| **Supplementary Tables** |  |
| Table S1. Summary table of quality control of DNAm data. | 7 |
| Table S2. Summary statistics of significant results in the discovery phase. | 8-9 |
| Table S3. Summary table of the validation and cross-tissue evaluation analyses. | 10 |
| Table S4. Summary statistics of stratified analyses based on asthma status. | 11 |
| Table S5. Summary statistics of stratified analyses based on albuterol use. | 12 |
| Table S6. Summary statistics of stratified analyses based on any controller medication use. | 13 |
| Table S7. Association of initial BDR status and albuterol-induced changes in DNAm. | 14 |
| Table S8. Summary results of the significant results in the meQTL analyses. | 15 |
| Table S9. Summary results of in silico eQTL analyses. | 16 |
| Table S10. Summary results of the eQTM analyses. | 17-19 |
| Table S11. Significant findings of the enrichment gene-set analysis. | 20-21 |
| Table S12. Summary results of DMRs identified with comb-p. | 22 |
| Table S13. Summary results of DMRs identified with *DMRcate*. | 23 |
| Table S14. Summary statistics of stratified analyses based on biological sex. | 24 |

**FIGURES**

**
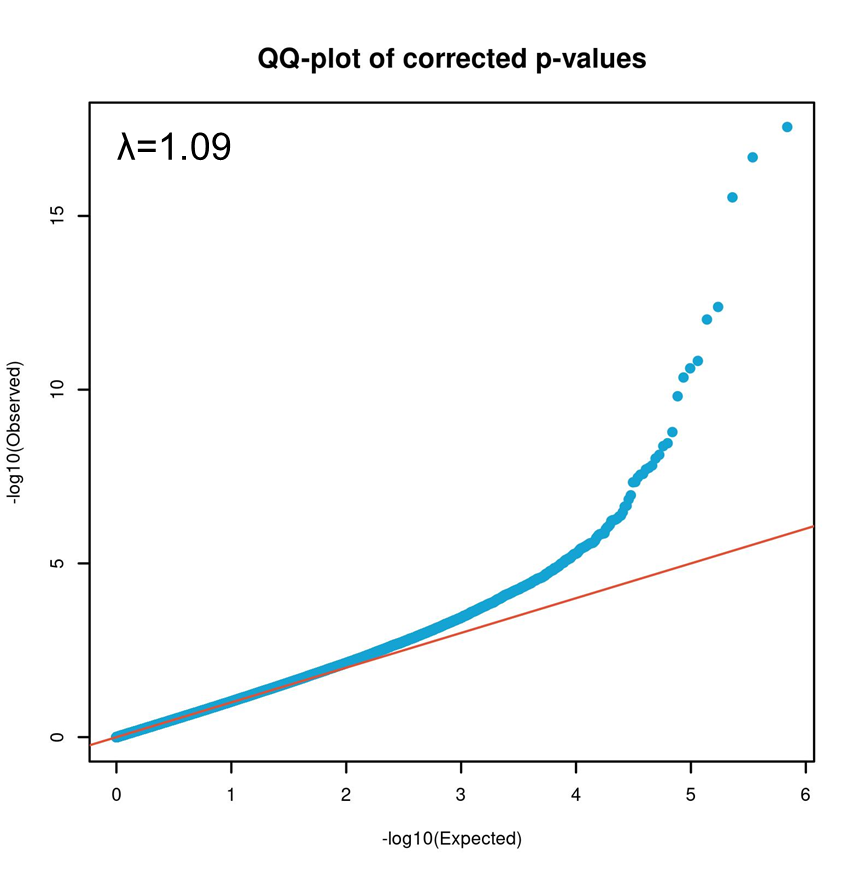
**

**Figure S1.** Quantile-quantile (Q-Q) plot of regression *p*-values of the EWAS of exposure to albuterol in nasal epithelial cells in the discovery phase. P-values were corrected using the *bacon* R package to control for bias and genomic inflation.

**
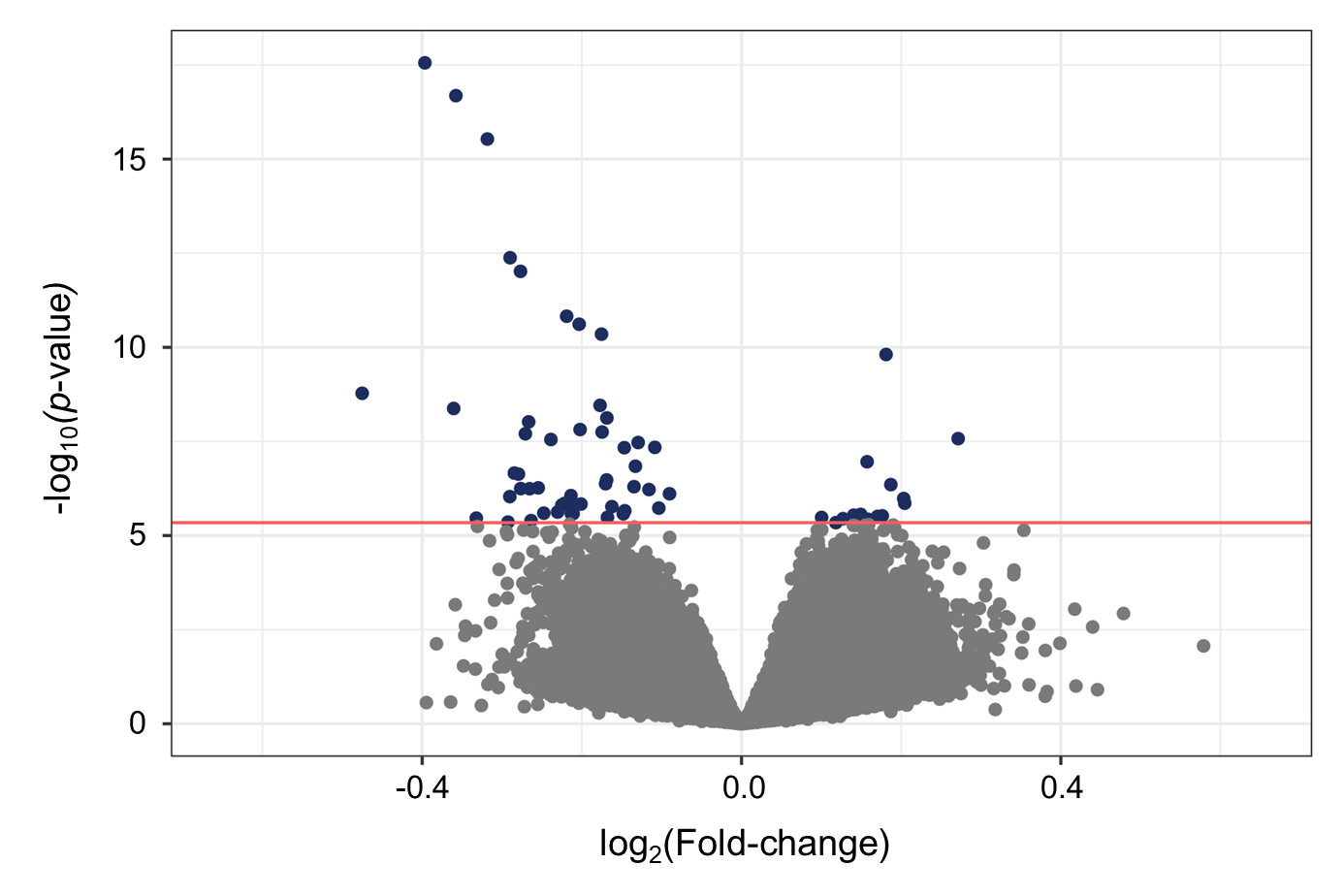
**

**Figure S2.** Volcano-plot of the EWAS of exposure to albuterol in nasal epithelial cells in the discovery phase. Each dot corresponds to a CpG site. The -log_10_(p-value) is represented along the y-axis and the effect is expressed as log_2_(Fold-change) on the x-axis. Negative log_2_(Fold-change) values correspond to CpGs hypomethylated after albuterol exposure, while positive values correspond to those hypermethylated. The red line represents the false discovery rate (FDR)<5% threshold.


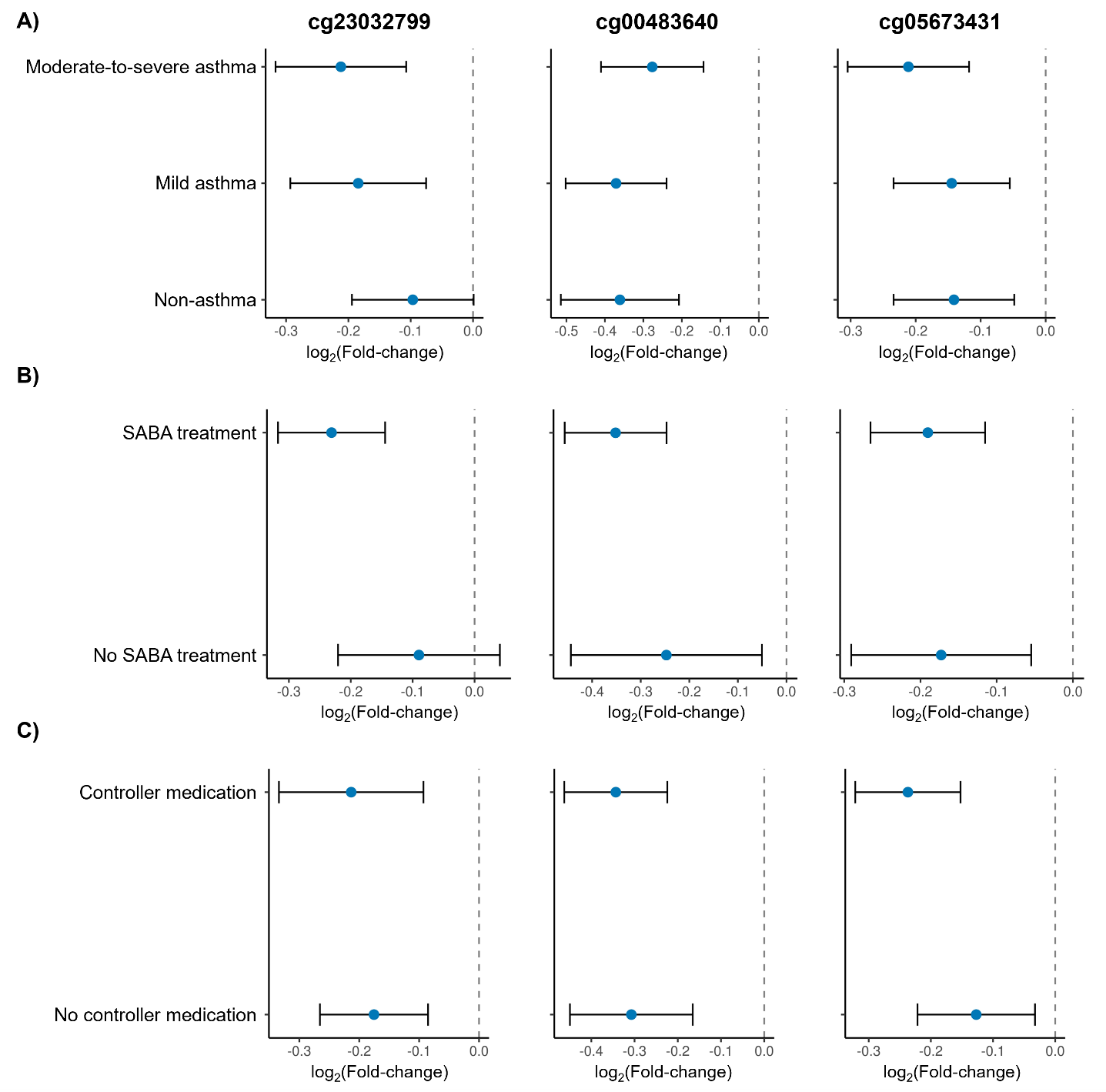


**Figure S3.** Forest plot of the stratified analyses by **A)** asthma status, **B)** receipt of SABA, and **C)** receipt of any controller medication of genome-wide significantly associated CpGs with evidence of validation. The effect expressed as log_2_(Fold-change) is represented along the x-axis. The dashed line indicates a null effect (log_2_Fold-change=0).


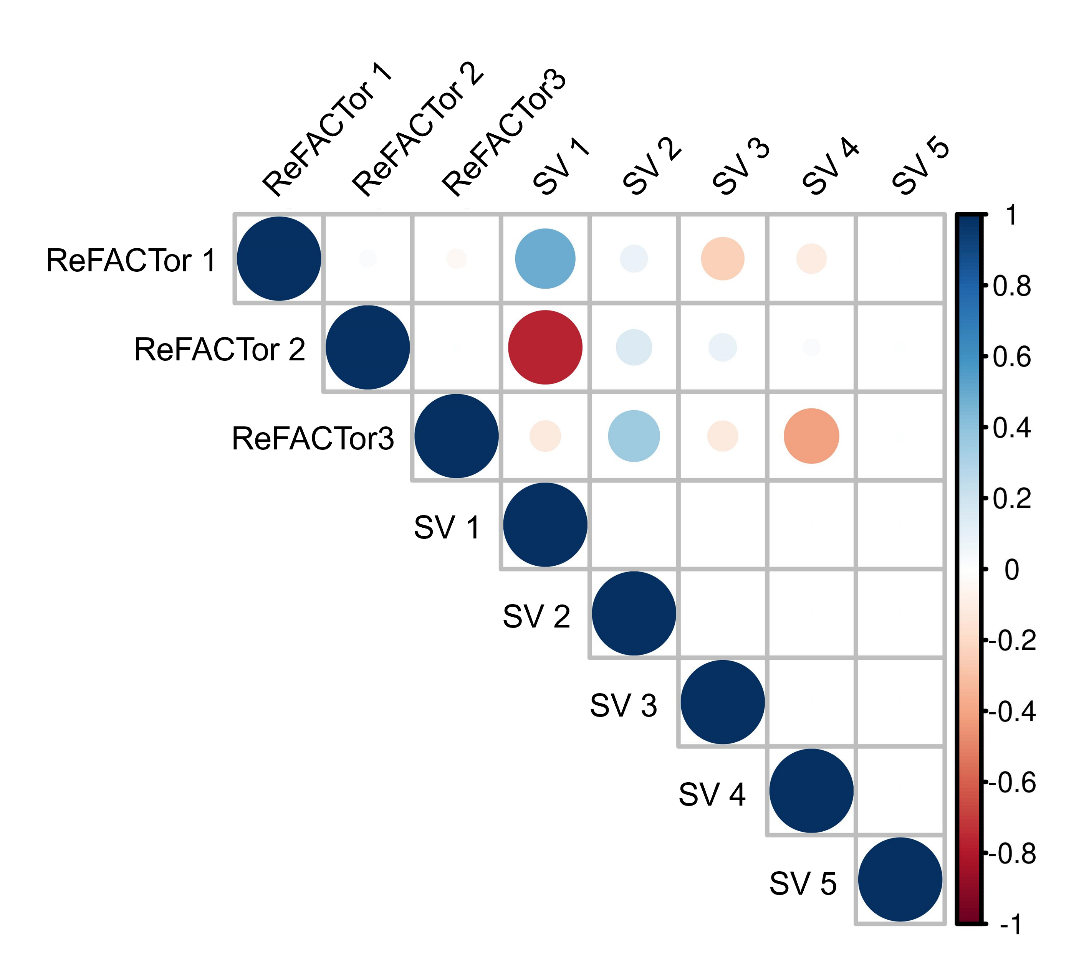


**Figure S4.** Correlation matrix plot of ReFACTor components estimated in DNAm data and significant surrogate variables (SV) identified in the gene expression data matrix. Correlations between each pair of variables are represented with circles (only significant correlations with a *p*<0.05 are represented). The size of the circles is proportional to the level of significance, with positive and negative correlations represented in blue and red, respectively. Only the SV-5 was not significantly correlated with any ReFACTor component.

**SUPPLEMENTARY TABLES**

| **Table S1**. Summary of the quality control of DNAm data. | | | |
| --- | --- | --- | --- |
|  | **GALA II** | **Obese Asthma Study** | |
|  | **NEC** | **NEC** | **BEC** |
| **Quality control of CpG sites** | | | |
| Initial number of CpGs | 856,553 | 856,553 | |
| Bad quality probes | 9,365 | 10,480 | |
| Probes on sexual chromosomes | 18,884 | 18,997 | |
| Cross-reactive probes | 43,254 | 43,254 | |
| Control probes genotyping SNPs | 59 | 59 | |
| Polymorphic probes | 11,755 | 6,265 | |
| Multimodal probes | 112,140 | 166,784 | 96,247 |
| Remaining CpGs | 689,483 | 622,699 | 693,236 |
| **Quality control of samples (paired samples)** | | | |
| Initial number of samples | 200 | 40 | 40 |
| Bad quality samples | 1 | 1 | 0 |
| Samples with sex discordance | 0 | 0 | 0 |
| High missingness rate | 0 | 0 | 0 |
| Potential cross-sample contaminated | 2 | 0 | 0 |
| Remaining non-paired samples | 97 | 10 | 10 |
| DNAm: DNA methylation; GALA II: Genes-environments & Admixture in Latino Americans Study; NEC: nasal epithelial cells; BEC: bronchial epithelial cells; SNPs: single nucleotide polymorphisms. | | | |

| **Table S2.** Summary table of CpGs associated with albuterol exposure in nasal samples with an FDR<0.05. | | | | | | | | | |
| --- | --- | --- | --- | --- | --- | --- | --- | --- | --- |
| **CpG** | **Chr** | **hg19** | **hg38** | **Gene** | **ΔDNAm (%)** | **logFC** | **SE** | ***p*-value** | **FDR** |
| cg10290200 | 7 | 128481295 | 128841241 | *FLNC* | -3.4 | -0.397 | 0.045 | 2.76x10^-18^ | 1.91x10^-12^ |
| cg00483640 | 6 | 2623483 | 2623249 | *MYLK4-LINC01600* | -4.9 | -0.358 | 0.042 | 2.05x10^-17^ | 7.07x10^-12^ |
| cg15833511 | 20 | 35256798 | 36628395 | *SLA2* | -15.3 | -0.318 | 0.039 | 2.88x10^-16^ | 6.64x10^-11^ |
| cg24254891 | 22 | 40395315 | 39999311 | *FAM83F* | -15.6 | -0.290 | 0.040 | 4.13x10^-13^ | 7.13x10^-8^ |
| cg12510044 | 22 | 22115473 | 21761184 | *MAPK1* | -9.1 | -0.277 | 0.039 | 9.46x10^-13^ | 1.31x10^-7^ |
| cg02424494 | 2 | 239200128 | 238291487 | *PER2* | -2.3 | -0.219 | 0.032 | 1.47x10^-11^ | 1.70x10^-6^ |
| cg03629778 | 7 | 129691325 | 130051485 | *ZC3HC1* | -10.1 | -0.203 | 0.030 | 2.45x10^-11^ | 2.42x10^-6^ |
| cg05673431 | 17 | 25871504 | 27544478 | *KSR1* | -2.4 | -0.175 | 0.027 | 4.54x10^-11^ | 3.93x10^-6^ |
| cg11023970 | 10 | 6337849 | 6295886 | *PFKFB3* | 4.4 | 0.181 | 0.028 | 1.56x10^-10^ | 1.20x10^-5^ |
| cg03956296 | 2 | 39951199 | 39724059 | *THUMPD2* | -17.7 | -0.475 | 0.079 | 1.69x10^-9^ | 1.17x10^-4^ |
| cg23032799 | 11 | 46296114 | 46274563 | *CREB3L1* | -6.5 | -0.177 | 0.030 | 3.50x10^-9^ | 2.20x10^-4^ |
| cg08161666 | 11 | 13034897 | 13013350 | *ARNTL* | -16.8 | -0.361 | 0.061 | 4.25x10^-9^ | 2.45x10^-4^ |
| cg10310427 | 6 | 30716985 | 30749208 | *IER3* | -8.2 | -0.169 | 0.029 | 7.56x10^-9^ | 4.02x10^-4^ |
| cg16769649 | 6 | 47326353 | 47358617 | *TNFRSF21* | -8.4 | -0.267 | 0.046 | 9.57x10^-9^ | 4.73x10^-4^ |
| cg26162522 | 1 | 57037116 | 56571443 | *PPAP2B* | -3.5 | -0.202 | 0.036 | 1.50x10^-8^ | 6.94x10^-4^ |
| cg16032470 | 20 | 31818199 | 33230393 | *BPIFA1* | -8.8 | -0.175 | 0.031 | 1.78x10^-8^ | 7.71x10^-4^ |
| cg03068616 | 6 | 6678498 | 6678265 | *LY86* | -12.6 | -0.271 | 0.048 | 1.98x10^-8^ | 8.05x10^-4^ |
| cg16519100 | 15 | 83869266 | 83200514 | *HDGFRP3* | 23.5 | 0.271 | 0.049 | 2.66x10^-8^ | 0.001 |
| cg22208174 | 9 | 109643142 | 106880861 | *ZNF462* | -7.6 | -0.239 | 0.043 | 2.80x10^-8^ | 0.001 |
| cg12845391 | 6 | 137545298 | 137224161 | *IFNGR1* | -4.4 | -0.129 | 0.023 | 3.45x10^-8^ | 0.001 |
| cg03946667 | 5 | 135394361 | 136058672 | *TGFBI* | -5.2 | -0.109 | 0.020 | 4.69x10^-8^ | 0.001 |
| cg12845808 | 5 | 141338604 | 141959039 | *PCDH12* | -7.3 | -0.147 | 0.027 | 4.71x10^-8^ | 0.001 |
| cg01083910 | 2 | 47537713 | 47310574 | *LOC101927043-EPCAM* | 6.6 | 0.157 | 0.030 | 1.10x10^-7^ | 0.003 |
| cg13172020 | 5 | 59042366 | 59746540 | *PDE4D* | -4.0 | -0.133 | 0.025 | 1.46x10^-7^ | 0.004 |
| cg20173976* | 18 | 55568547 | 57901315 | *ATP8B1* | -12.8 | -0.285 | 0.055 | 2.20x10^-7^ | 0.006 |
| cg07907216 | 4 | 109862988 | 108941832 | *COL25A1* | -1.6 | -0.279 | 0.054 | 2.34x10^-7^ | 0.006 |
| cg10271993* | 8 | 110593242 | 109581013 | *SYBU* | -7.4 | -0.169 | 0.033 | 3.30x10^-7^ | 0.008 |
| cg25243766 | 11 | 123322182 | 123451474 | *GRAMD1B* | -8.3 | -0.170 | 0.034 | 4.15x10^-7^ | 0.010 |
| cg05284643 | 1 | 205215690 | 205246562 | *TMCC2* | 1.5 | 0.187 | 0.037 | 4.39x10^-7^ | 0.010 |
| cg13928566 | 12 | 130624596 | 130140051 | *FZD10* | -6.8 | -0.135 | 0.027 | 5.08x10^-7^ | 0.012 |
| cg21334677 | 17 | 4691477 | 4788182 | *GLTPD2* | -11.0 | -0.255 | 0.051 | 5.43x10^-7^ | 0.012 |
| cg00901816 | 15 | 27656099 | 27410953 | *GABRG3* | -3.9 | -0.277 | 0.055 | 5.68x10^-7^ | 0.012 |
| cg15589042 | 17 | 4691376 | 4788081 | *GLTPD2* | -11.6 | -0.265 | 0.053 | 5.72x10^-7^ | 0.012 |
| cg03995830 | 10 | 14782991 | 14740992 | *FAM107B* | -4.3 | -0.116 | 0.023 | 6.11x10^-7^ | 0.012 |
| cg15633082 | 7 | 157840336 | 158047644 | *PTPRN2* | -1.7 | -0.090 | 0.018 | 8.06x10^-7^ | 0.016 |
| cg03525786 | 5 | 76085956 | 76790131 | *F2RL1* | -9.8 | -0.214 | 0.043 | 8.68x10^-7^ | 0.017 |
| cg23207054 | 17 | 38171530 | 40015277 | *CSF3* | -11.9 | -0.290 | 0.059 | 9.29x10^-7^ | 0.017 |
| cg02750308 | 4 | 113559408 | 112638252 | *LARP7-ZGRF1* | 3.0 | 0.203 | 0.042 | 1.03x10^-6^ | 0.019 |
| cg08557669 | 11 | 63626186 | 63858714 | *MARK2* | -9.8 | -0.215 | 0.044 | 1.36x10^-6^ | 0.023 |
| cg04038943 | 1 | 46196297 | 45730625 | *IPP* | 18.5 | 0.204 | 0.042 | 1.38x10^-6^ | 0.023 |
| cg08834429 | 16 | 67694862 | 67660959 | *ACD-PARD6A* | -9.6 | -0.222 | 0.046 | 1.41x10^-6^ | 0.023 |
| cg13030011 | 7 | 23450804 | 23411185 | *IGF2BP3* | -1.2 | -0.212 | 0.044 | 1.43x10^-6^ | 0.023 |
| cg04122198 | 2 | 159174180 | 158317668 | *CCDC148* | -6.1 | -0.201 | 0.042 | 1.45x10^-6^ | 0.023 |
| cg18202623* | 4 | 126236316 | 125315161 | *FAT4* | -9.5 | -0.225 | 0.047 | 1.55x10^-6^ | 0.024 |
| cg09247963 | 12 | 30907923 | 30754989 | *CAPRIN2* | -6.7 | -0.162 | 0.034 | 1.69x10^-6^ | 0.026 |
| cg26175287* | 14 | 95877009 | 95410672 | *C14orf139* | -3.4 | -0.104 | 0.022 | 1.90x10^-6^ | 0.029 |
| cg15427166 | 2 | 134084354 | 133326782 | *MIR7853-NCKAP5* | -3.8 | -0.146 | 0.031 | 2.20x10^-6^ | 0.032 |
| cg10194812 | 10 | 95360612 | 93600855 | *RBP4* | -10.0 | -0.230 | 0.049 | 2.38x10^-6^ | 0.034 |
| cg25562233 | 22 | 38564163 | 38168156 | *PLA2G6* | -10.5 | -0.248 | 0.053 | 2.55x10^-6^ | 0.035 |
| cg13174688* | 18 | 8801435 | 8801437 | *MTCL1* | -8.4 | -0.213 | 0.045 | 2.60x10^-6^ | 0.035 |
| cg25145977* | 11 | 82708637 | 82997595 | *RAB30* | -10.5 | -0.211 | 0.045 | 2.60x10^-6^ | 0.035 |
| cg15479068* | 22 | 33454444 | 33058458 | *SYN3* | -7.2 | -0.148 | 0.032 | 2.65x10^-6^ | 0.035 |
| cg22805990 | 7 | 30634502 | 30594886 | *GARS* | 12.3 | 0.149 | 0.032 | 2.73x10^-6^ | 0.036 |
| cg07033577 | 17 | 55540785 | 57463424 | *MSI2* | 3.1 | 0.141 | 0.030 | 2.87x10^-6^ | 0.037 |
| cg11225759 | 15 | 92614493 | 92071263 | *SLCO3A1* | 2.6 | 0.176 | 0.038 | 2.97x10^-6^ | 0.037 |
| cg05122299 | 16 | 10693569 | 10599712 | *EMP2* | 0.9 | 0.170 | 0.037 | 3.07x10^-6^ | 0.038 |
| cg17388916 | 9 | 131370263 | 128607984 | *MIR1268A-SPTAN1* | -8.1 | -0.168 | 0.036 | 3.26x10^-6^ | 0.040 |
| cg00047079 | 13 | 79188975 | 78614840 | *RNF219* | 2.5 | 0.100 | 0.022 | 3.37x10^-6^ | 0.040 |
| cg16540215 | 11 | 43856925 | 43835375 | *HSD17B12* | -12.0 | -0.332 | 0.072 | 3.47x10^-6^ | 0.041 |
| cg02733432 | 10 | 126379645 | 124691076 | *FAM53B* | 1.8 | 0.127 | 0.027 | 3.58x10^-6^ | 0.041 |
| cg26545845 | 10 | 99175095 | 97415338 | *PGAM1* | 1.9 | 0.140 | 0.030 | 3.64x10^-6^ | 0.041 |
| cg11299602 | 3 | 150398505 | 150680718 | *ERICH6* | 1.5 | 0.158 | 0.034 | 3.66x10^-6^ | 0.041 |
| cg22295628 | 17 | 1733433 | 1830139 | *RPA1-SMYD4* | 11.1 | 0.137 | 0.030 | 3.81x10^-6^ | 0.042 |
| cg01830886 | 1 | 39636619 | 39170947 | *MACF1* | -11.3 | -0.264 | 0.057 | 4.03x10^-6^ | 0.044 |
| cg27268936 | 6 | 82796449 | 82086732 | *IBTK* | -3.2 | -0.293 | 0.064 | 4.42x10^-6^ | 0.047 |
| cg09589728 | 12 | 96439560 | 96045782 | *LTA4H* | 3.3 | 0.118 | 0.026 | 4.58x10^-6^ | 0.048 |
| *CpGs with a ΔDNAm<2%. Genomic positions are indicated in hg19/GRCh37 and hg38/GRCh38 Genome Assemblies. Chr: chromosome; logFC: log_2_(fold-change); SE: standard error; FDR: false discovery rate. | | | | | | | | | |

| **Table S3.** Summary results of the validation in bronchial epithelial cells from independent donors. | | | | | | | |
| --- | --- | --- | --- | --- | --- | --- | --- |
|  |  |  |  | **Nasal epithelia** | | **Bronchial epithelia** | |
| **CpG** | **Chr** | **Position*** | **Gene** | **logFC** | ***p*-value** | **logFC** | ***p*-value** |
| cg23032799 | 11 | 46274563 | *CREB3L1* | -0.264 | 1.53x10^-4^ | -0.169 | 0.030 |
| cg00483640 | 6 | 2623249 | *MYLK4-LINC01600* | -0.321 | 0.003 | -0.146 | 0.184 |
| cg05673431 | 17 | 27544478 | *KSR1* | -0.240 | 0.004 | -0.063 | 0.384 |
| *Genomic positions are indicated in hg38/GRCh38 Genome Assembly. Chr: chromosome; logFC: log_2_(fold-change). | | | | | | | |

| **Table S4**. Summary table of heterogeneity assessment on stratified analyses by asthma status. | | | | | | | | | | | | | | |
| --- | --- | --- | --- | --- | --- | --- | --- | --- | --- | --- | --- | --- | --- | --- |
|  |  |  |  | **Non-asthma (n=33)** | | | **Mild asthma (n=31)** | | | **Severe asthma (n=36)** | | | **Meta-analysis** | |
| **CpG** | **Gene** | **Chr** | **Position*** | **logFC** | **SE** | ***p*** | **logFC** | **SE** | ***p*** | **logFC** | **SE** | ***p*** | **I^2^** | **Q-*p*** |
| cg10290200 | *FLNC* | 7 | 128841241 | -0.37 | 0.07 | 1.07x10-^5^ | -0.32 | 0.09 | 0.001 | -0.44 | 0.07 | 5.43x10^-8^ | 0 | 0.508 |
| cg00483640 | *MYLK4-LINC01600* | 6 | 2623249 | -0.36 | 0.08 | 3.85x10^-5^ | -0.37 | 0.06 | 2.17x10^-6^ | -0.28 | 0.07 | 1.57x10^-4^ | 0 | 0.546 |
| cg15833511 | *SLA2* | 20 | 36628395 | -0.32 | 0.06 | 5.81x10^-6^ | -0.24 | 0.07 | 0.001 | -0.38 | 0.07 | 2.58x10^-6^ | 7.67 | 0.339 |
| cg24254891 | *FAM83F* | 22 | 39999311 | -0.27 | 0.05 | 2.49x10^-5^ | -0.20 | 0.08 | 0.018 | -0.31 | 0.05 | 1.54x10^-6^ | 0 | 0.533 |
| cg12510044 | *MAPK1* | 22 | 21761184 | -0.27 | 0.06 | 1.56x10^-4^ | -0.21 | 0.06 | 0.002 | -0.27 | 0.06 | 1.51x10^-4^ | 0 | 0.724 |
| cg02424494 | *PER2* | 2 | 238291487 | -0.18 | 0.06 | 0.003 | -0.21 | 0.05 | 2.87x10^-4^ | -0.24 | 0.05 | 9.81x10^-5^ | 0 | 0.736 |
| cg03629778 | *ZC3HC1* | 7 | 130051485 | -0.16 | 0.06 | 0.007 | -0.31 | 0.05 | 1.47x10^-7^ | -0.18 | 0.04 | 2.47x10^-4^ | 64.94 | 0.058 |
| cg05673431 | *KSR1* | 17 | 27544478 | -0.14 | 0.05 | 0.004 | -0.14 | 0.04 | 0.002 | -0.21 | 0.05 | 5.06x10^-5^ | 0 | 0.473 |
| cg11023970 | *PFKFB3* | 10 | 6295886 | 0.19 | 0.05 | 2.59x10^-4^ | 0.16 | 0.05 | 0.005 | 0.21 | 0.05 | 1.21x10^-4^ | 0 | 0.834 |
| cg03956296 | *THUMPD2* | 2 | 39724059 | -0.42 | 0.14 | 0.004 | -0.33 | 0.11 | 0.005 | -0.68 | 0.12 | 9.55x10^-7^ | 61.39 | 0.075 |
| cg23032799 | *CREB3L1* | 11 | 46274563 | -0.10 | 0.05 | 0.052 | -0.18 | 0.05 | 0.002 | -0.21 | 0.05 | 2.18x10^-4^ | 32.57 | 0.227 |
| cg08161666 | *ARNTL* | 11 | 13013350 | -0.28 | 0.09 | 0.003 | -0.36 | 0.11 | 0.003 | -0.40 | 0.09 | 1.49x10^-4^ | 0 | 0.631 |
| cg10310427 | *IER3* | 6 | 30749208 | -0.19 | 0.05 | 3.90x10^-4^ | -0.15 | 0.05 | 0.005 | -0.11 | 0.05 | 0.024 | 0 | 0.481 |
| cg16769649 | *TNFRSF21* | 6 | 47358617 | -0.17 | 0.08 | 0.035 | -0.34 | 0.07 | 6.70x10^-5^ | -0.23 | 0.07 | 0.003 | 23.35 | 0.271 |
| cg26162522 | *PPAP2B* | 1 | 56571443 | -0.22 | 0.07 | 0.003 | -0.11 | 0.05 | 0.030 | -0.22 | 0.06 | 3.72x10^-4^ | 27.75 | 0.251 |
| cg16032470 | *BPIFA1* | 20 | 33230393 | -0.11 | 0.04 | 0.013 | -0.16 | 0.06 | 0.008 | -0.22 | 0.05 | 3.02x10^-4^ | 13.26 | 0.316 |
| cg03068616 | *LY86* | 6 | 6678265 | -0.24 | 0.06 | 2.85x10^-4^ | -0.17 | 0.10 | 0.090 | -0.34 | 0.07 | 3.27x10^-5^ | 9.99 | 0.329 |
| cg16519100 | *HDGFRP3* | 15 | 83200514 | 0.24 | 0.07 | 0.002 | 0.26 | 0.08 | 0.004 | 0.34 | 0.08 | 1.36x10^-4^ | 0 | 0.607 |
| cg22208174 | *ZNF462* | 9 | 106880861 | -0.19 | 0.09 | 0.033 | -0.23 | 0.06 | 0.001 | -0.23 | 0.06 | 0.001 | 0 | 0.916 |
| cg12845391 | *IFNGR1* | 6 | 137224161 | -0.06 | 0.04 | 0.112 | -0.20 | 0.05 | 2.36x10^-4^ | -0.13 | 0.04 | 0.003 | 60.24 | 0.081 |
| cg12845808 | *PCDH12* | 5 | 141959039 | -0.11 | 0.03 | 0.002 | -0.05 | 0.04 | 0.272 | -0.14 | 0.03 | 8.36x10^-5^ | 41.17 | 0.183 |
| cg03946667 | *TGFBI* | 5 | 136058672 | -0.12 | 0.04 | 0.008 | -0.13 | 0.06 | 0.025 | -0.15 | 0.04 | 0.001 | 0 | 0.850 |
| *Genomic positions are indicated in hg38/GRCh38 Genome Assembly. Chr: chromosome; logFC: log_2_(fold-change); SE: standard error; Q-*p*: Cochran's Q *p*-value. | | | | | | | | | | | | | | |

| **Table S5**. Summary table of heterogeneity assessment on stratified analyses by SABA treatment. | | | | | | | | | | | |
| --- | --- | --- | --- | --- | --- | --- | --- | --- | --- | --- | --- |
|  |  |  |  | **SABA-naive patients (n=15)** | | | **Treated with SABA (n=52)** | | | **Meta-analysis** | |
| **CpG** | **Gene** | **Chr** | **Position*** | **logFC** | **SE** | ***p*** | **logFC** | **SE** | ***p*** | **I^2^** | **Q-*p*** |
| cg10290200 | *FLNC* | 7 | 128841241 | -0.32 | 0.10 | 0.005 | -0.43 | 0.06 | 5.51x10^-9^ | 0 | 0.335 |
| cg00483640 | *MYLK4-LINC01600* | 6 | 2623249 | -0.25 | 0.09 | 0.017 | -0.35 | 0.05 | 1.25x10^-8^ | 0 | 0.326 |
| cg15833511 | *SLA2* | 20 | 36628395 | -0.11 | 0.09 | 0.221 | -0.39 | 0.05 | 1.35x10^-9^ | 86.28 | 0.007 |
| cg24254891 | *FAM83F* | 22 | 39999311 | -0.32 | 0.09 | 0.002 | -0.27 | 0.06 | 2.57x10^-5^ | 0 | 0.600 |
| cg12510044 | *MAPK1* | 22 | 21761184 | -0.29 | 0.08 | 0.002 | -0.24 | 0.05 | 2.83x10^-5^ | 0 | 0.599 |
| cg02424494 | *PER2* | 2 | 238291487 | -0.13 | 0.07 | 0.080 | -0.25 | 0.04 | 4.43x10^-7^ | 47.35 | 0.168 |
| cg03629778 | *ZC3HC1* | 7 | 130051485 | -0.19 | 0.07 | 0.012 | -0.24 | 0.04 | 3.05x10^-8^ | 0 | 0.543 |
| cg05673431 | *KSR1* | 17 | 27544478 | -0.17 | 0.06 | 0.007 | -0.19 | 0.04 | 5.00x10^-6^ | 0 | 0.794 |
| cg11023970 | *PFKFB3* | 10 | 6295886 | 0.10 | 0.07 | 0.182 | 0.20 | 0.04 | 2.41x10^-6^ | 39.81 | 0.197 |
| cg03956296 | *THUMPD2* | 2 | 39724059 | -0.35 | 0.13 | 0.017 | -0.58 | 0.10 | 2.98x10^-7^ | 46.03 | 0.173 |
| cg23032799 | *CREB3L1* | 11 | 46274563 | -0.09 | 0.06 | 0.165 | -0.23 | 0.04 | 1.87x10^-6^ | 71.57 | 0.061 |
| cg08161666 | *ARNTL* | 11 | 13013350 | -0.32 | 0.17 | 0.076 | -0.40 | 0.08 | 5.20x10^-6^ | 0 | 0.634 |
| cg10310427 | *IER3* | 6 | 30749208 | -0.08 | 0.07 | 0.257 | -0.17 | 0.04 | 1.44x10^-4^ | 28.13 | 0.238 |
| cg16769649 | *TNFRSF21* | 6 | 47358617 | -0.25 | 0.10 | 0.028 | -0.31 | 0.06 | 4.24x10^-6^ | 0 | 0.590 |
| cg26162522 | *PPAP2B* | 1 | 56571443 | -0.14 | 0.08 | 0.109 | -0.17 | 0.04 | 1.88x10^-4^ | 0 | 0.730 |
| cg16032470 | *BPIFA1* | 20 | 33230393 | -0.08 | 0.07 | 0.292 | -0.23 | 0.05 | 7.42x10^-6^ | 69.79 | 0.069 |
| cg03068616 | *LY86* | 6 | 6678265 | -0.17 | 0.11 | 0.146 | -0.30 | 0.07 | 5.82x10^-5^ | 4.62 | 0.306 |
| cg16519100 | *HDGFRP3* | 15 | 83200514 | 0.18 | 0.11 | 0.105 | 0.34 | 0.07 | 2.77x10^-6^ | 40.92 | 0.193 |
| cg22208174 | *ZNF462* | 9 | 106880861 | -0.16 | 0.10 | 0.110 | -0.25 | 0.05 | 1.37x10^-6^ | 0 | 0.394 |
| cg12845391 | *IFNGR1* | 6 | 137224161 | -0.17 | 0.06 | 0.017 | -0.14 | 0.03 | 1.13x10^-4^ | 0 | 0.706 |
| cg12845808 | *PCDH12* | 5 | 141959039 | -0.03 | 0.07 | 0.647 | -0.11 | 0.03 | 2.19x10^-4^ | 12.32 | 0.286 |
| cg03946667 | *TGFBI* | 5 | 136058672 | -0.08 | 0.07 | 0.262 | -0.18 | 0.04 | 3.98x10^-5^ | 35.49 | 0.213 |
| *Genomic positions are indicated in hg38/GRCh38 Genome Assembly. SABA: short-acting beta-agonists; Chr: chromosome; logFC: log_2_(fold-change); SE: standard error; Q-*p*: Cochran's Q *p*-value. | | | | | | | | | | | |

| **Table S6**. Summary table of heterogeneity assessment on stratified analyses by controller medication use. | | | | | | | | | | | |
| --- | --- | --- | --- | --- | --- | --- | --- | --- | --- | --- | --- |
|  |  |  |  | **No controller (n=35)*** | | | **Any controller (n=32)*** | | | **Meta-analysis** | |
| **CpG** | **Gene** | **Chr** | **Position**^†^ | **logFC** | **SE** | ***p*** | **logFC** | **SE** | ***p*** | **I^2^** | **Q-*p*** |
| cg10290200 | *FLNC* | 7 | 128841241 | -0.39 | 0.08 | 2.34x10^-5^ | -0.38 | 0.07 | 2.80x10^-6^ | 0 | 0.956 |
| cg00483640 | *MYLK4-LINC01600* | 6 | 2623249 | -0.31 | 0.07 | 9.59x10^-5^ | -0.34 | 0.06 | 1.46x10^-6^ | 0 | 0.694 |
| cg15833511 | *SLA2* | 20 | 36628395 | -0.29 | 0.06 | 1.27x10^-5^ | -0.34 | 0.08 | 1.15x10^-4^ | 0 | 0.554 |
| cg24254891 | *FAM83F* | 22 | 39999311 | -0.22 | 0.08 | 0.006 | -0.31 | 0.05 | 1.55x10^-6^ | 4.63 | 0.306 |
| cg12510044 | *MAPK1* | 22 | 21761184 | -0.22 | 0.05 | 1.95x10^-4^ | -0.27 | 0.07 | 0.001 | 0 | 0.571 |
| cg02424494 | *PER2* | 2 | 238291487 | -0.22 | 0.05 | 1.57x10^-4^ | -0.23 | 0.06 | 2.99x10^-4^ | 0 | 0.909 |
| cg03629778 | *ZC3HC1* | 7 | 130051485 | -0.24 | 0.04 | 3.67x10^-6^ | -0.22 | 0.05 | 1.31x10^-4^ | 0 | 0.713 |
| cg05673431 | *KSR1* | 17 | 27544478 | -0.13 | 0.05 | 0.010 | -0.24 | 0.04 | 2.34x10^-6^ | 67.71 | 0.078 |
| cg11023970 | *PFKFB3* | 10 | 6295886 | 0.17 | 0.06 | 0.004 | 0.20 | 0.04 | 4.42x10^-5^ | 0 | 0.709 |
| cg03956296 | *THUMPD2* | 2 | 39724059 | -0.37 | 0.12 | 0.004 | -0.70 | 0.11 | 2.00x10^-7^ | 76.71 | 0.038 |
| cg23032799 | *CREB3L1* | 11 | 46274563 | -0.18 | 0.04 | 3.57x10^-4^ | -0.21 | 0.06 | 0.001 | 0 | 0.608 |
| cg08161666 | *ARNTL* | 11 | 13013350 | -0.39 | 0.10 | 2.48x10^-4^ | -0.39 | 0.11 | 0.001 | 0 | 0.998 |
| cg10310427 | *IER3* | 6 | 30749208 | -0.11 | 0.05 | 0.022 | -0.16 | 0.05 | 0.004 | 0 | 0.530 |
| cg16769649 | *TNFRSF21* | 6 | 47358617 | -0.36 | 0.06 | 2.15x10^-6^ | -0.19 | 0.08 | 0.022 | 64.19 | 0.095 |
| cg26162522 | *PPAP2B* | 1 | 56571443 | -0.13 | 0.05 | 0.013 | -0.21 | 0.06 | 0.001 | 9.87 | 0.292 |
| cg16032470 | *BPIFA1* | 20 | 33230393 | -0.13 | 0.05 | 0.019 | -0.24 | 0.05 | 9.31x10^-5^ | 53.14 | 0.144 |
| cg03068616 | *LY86* | 6 | 6678265 | -0.14 | 0.08 | 0.098 | -0.39 | 0.07 | 9.54x10^-6^ | 78.83 | 0.030 |
| cg16519100 | *HDGFRP3* | 15 | 83200514 | 0.35 | 0.07 | 4.44x10^-5^ | 0.26 | 0.08 | 0.003 | 0 | 0.452 |
| cg22208174 | *ZNF462* | 9 | 106880861 | -0.22 | 0.06 | 4.35x10^-4^ | -0.24 | 0.06 | 3.61x10^-4^ | 0 | 0.801 |
| cg12845391 | *IFNGR1* | 6 | 137224161 | -0.16 | 0.04 | 4.55x10^-4^ | -0.14 | 0.04 | 0.003 | 0 | 0.760 |
| cg12845808 | *PCDH12* | 5 | 141959039 | -0.06 | 0.04 | 0.120 | -0.13 | 0.03 | 0.001 | 31.08 | 0.228 |
| cg03946667 | *TGFBI* | 5 | 136058672 | -0.17 | 0.05 | 0.001 | -0.11 | 0.05 | 0.026 | 6.38 | 0.301 |
| *Based on the prescription of inhaled corticosteroids, long-acting beta-agonists, combo medication, leukotriene receptor antagonists, oral corticosteroids, and/or theophylline. ^†^Genomic positions are indicated in hg38/GRCh38 Genome Assembly. Chr: chromosome; logFC: log_2_(fold-change); SE: standard error; Q-*p*: Cochran's Q *p*-value. | | | | | | | | | | | |

| **Table S7.** Association of initial BDR status and albuterol-induced changes in DNAm. | | | | | | | |
| --- | --- | --- | --- | --- | --- | --- | --- |
| **CpG** | **Chr** | **Position*** | **Gene** | **Coefficient** | **SE** | ***p*-value** | **FDR** |
| cg23032799 | 11 | 46274563 | *CREB3L1* | 0.03 | 0.01 | 0.004 | 0.089 |
| cg12510044 | 22 | 21761184 | *MAPK1* | 0.03 | 0.01 | 0.025 | 0.260 |
| cg08161666 | 11 | 13013350 | *ARNTL* | 0.03 | 0.01 | 0.035 | 0.260 |
| cg11023970 | 10 | 6295886 | *PFKFB3* | 0.02 | 0.01 | 0.222 | 0.851 |
| cg12845808 | 5 | 141959039 | *PCDH12* | 0.01 | 0.01 | 0.317 | 0.851 |
| cg16769649 | 6 | 47358617 | *TNFRSF21* | 0.01 | 0.01 | 0.339 | 0.851 |
| cg03068616 | 6 | 6678265 | *LY86* | 0.01 | 0.01 | 0.352 | 0.851 |
| cg26162522 | 1 | 56571443 | *PPAP2B* | -0.01 | 0.01 | 0.358 | 0.851 |
| cg10290200 | 7 | 128841241 | *FLNC* | -0.01 | 0.01 | 0.393 | 0.851 |
| cg03956296 | 2 | 39724059 | *THUMPD2* | -0.01 | 0.01 | 0.401 | 0.851 |
| cg02424494 | 2 | 238291487 | *PER2* | 0.01 | 0.01 | 0.445 | 0.851 |
| cg05673431 | 17 | 27544478 | *KSR1* | -0.01 | 0.01 | 0.464 | 0.851 |
| cg16032470 | 20 | 33230393 | *BPIFA1* | -0.01 | 0.01 | 0.522 | 0.857 |
| cg10310427 | 6 | 30749208 | *IER3* | 0.01 | 0.01 | 0.546 | 0.857 |
| cg24254891 | 22 | 39999311 | *FAM83F* | -0.01 | 0.01 | 0.685 | 0.949 |
| cg03629778 | 7 | 130051485 | *ZC3HC1* | 0.01 | 0.01 | 0.690 | 0.949 |
| cg22208174 | 9 | 106880861 | *ZNF462* | 4.02x10^-3^ | 0.01 | 0.750 | 0.960 |
| cg03946667 | 5 | 136058672 | *TGFBI* | -3.05x10^-3^ | 0.01 | 0.802 | 0.960 |
| cg12845391 | 6 | 137224161 | *IFNGR1* | 2.48x10^-3^ | 0.01 | 0.849 | 0.960 |
| cg16519100 | 15 | 83200514 | *HDGFRP3* | -2.27x10^-3^ | 0.01 | 0.872 | 0.960 |
| cg00483640 | 6 | 2623249 | *MYLK4-LINC01600* | -1.06x10^-3^ | 0.01 | 0.930 | 0.974 |
| cg15833511 | 20 | 36628395 | *SLA2* | -3.71x10^-4^ | 0.01 | 0.977 | 0.977 |
| *Genomic positions are indicated in hg38/GRCh38 Genome Assembly. Chr: chromosome; SE: standard error; FDR: false discovery rate. | | | | | | | |

| **Table S8.** Summary table of the significant results in the meQTL analyses. | | | | | | | | | | | | | | | |
| --- | --- | --- | --- | --- | --- | --- | --- | --- | --- | --- | --- | --- | --- | --- | --- |
| **rsID** | **Chr** | **Position*** | **Distance (bp)** | | | | **A1** | **A2** | **MAF** | **Coefficient** | | **SE** | ***p*-value** | | **FDR** |
| **cg23032799 (*CREB3L1*)** | | | | | | | | | | | | | | | |
| rs11038897 | 11 | 46449277 | 174714 | | | | T | C | 0.281 | 0.491 | | 0.134 | 4.24x10^-4^ | | 0.047 |
| rs11038900 | 11 | 46473752 | 199189 | | | | G | A | 0.286 | 0.477 | | 0.133 | 5.49x10^-4^ | | 0.047 |
| rs12578042 | 11 | 46493454 | 218891 | | | | G | C | 0.286 | 0.477 | | 0.133 | 5.49x10^-4^ | | 0.047 |
| rs16938506 | 11 | 46432763 | 158200 | | | | C | A | 0.286 | 0.477 | | 0.133 | 5.49x10^-4^ | | 0.047 |
| rs3802888 | 11 | 46494352 | 219789 | | | | G | A | 0.286 | 0.477 | | 0.133 | 5.49x10^-4^ | | 0.047 |
| rs6485682 | 11 | 46424714 | 150151 | | | | C | A | 0.286 | 0.477 | | 0.133 | 5.49x10^-4^ | | 0.047 |
| rs7130141 | 11 | 46478324 | 203761 | | | | T | C | 0.286 | 0.477 | | 0.133 | 5.49x10^-4^ | | 0.047 |
| **cg00483640 (*MYLK4-LINC01600*)** | | | | | | | | | | | | | | | |
| rs6901966 | 6 | 2623299 | 50 | | | | C | G | 0.245 | -0.757 | | 0.128 | 6.83x10^-8^ | | 1.20x10^-4^ |
| rs9391958 | 6 | 2628165 | 4916 | | | | T | C | 0.208 | -0.663 | | 0.142 | 1.10x10^-5^ | | 0.002 |
| rs7757270 | 6 | 2617085 | -6164 | | | | C | A | 0.203 | -0.660 | | 0.142 | 1.21x10^-5^ | | 0.002 |
| rs13196773 | 6 | 2627106 | 3857 | | | | T | C | 0.203 | -0.660 | | 0.142 | 1.21x10^-5^ | | 0.002 |
| rs4959690 | 6 | 2618908 | -4341 | | | | T | C | 0.203 | -0.660 | | 0.142 | 1.21x10^-5^ | | 0.002 |
| rs6902511 | 6 | 2623586 | 337 | | | | A | G | 0.203 | -0.660 | | 0.142 | 1.21x10^-5^ | | 0.002 |
| rs6915706 | 6 | 2625510 | 2261 | | | | T | C | 0.203 | -0.660 | | 0.142 | 1.21x10^-5^ | | 0.002 |
| rs6929016 | 6 | 2615643 | -7606 | | | | C | G | 0.203 | -0.660 | | 0.142 | 1.21x10^-5^ | | 0.002 |
| rs6932178 | 6 | 2618538 | -4711 | | | | C | T | 0.203 | -0.660 | | 0.142 | 1.21x10^-5^ | | 0.002 |
| rs6938328 | 6 | 2629259 | 6010 | | | | A | G | 0.203 | -0.660 | | 0.142 | 1.21x10^-5^ | | 0.002 |
| rs9378338 | 6 | 2619537 | -3712 | | | | T | C | 0.203 | -0.660 | | 0.142 | 1.21x10^-5^ | | 0.002 |
| rs9378720 | 6 | 2624140 | 891 | | | | A | G | 0.203 | -0.660 | | 0.142 | 1.21x10^-5^ | | 0.002 |
| rs6931606 | 6 | 2618218 | -5031 | | | | A | T | 0.208 | -0.657 | | 0.142 | 1.38x10^-5^ | | 0.002 |
| rs6931607 | 6 | 2618219 | -5030 | | | | C | T | 0.208 | -0.657 | | 0.142 | 1.38x10^-5^ | | 0.002 |
| rs6903030 | 6 | 2608201 | -15048 | | | | C | T | 0.473 | -0.515 | | 0.128 | 1.33x10^-4^ | | 0.016 |
| rs9392412 | 6 | 2652994 | 29745 | | | | T | C | 0.245 | -0.582 | | 0.146 | 1.43x10^-4^ | | 0.016 |
| rs1545853 | 6 | 2642766 | 19517 | | | | A | G | 0.245 | -0.582 | | 0.148 | 1.71x10^-4^ | | 0.017 |
| rs9378722 | 6 | 2653266 | 30017 | | | | A | G | 0.245 | -0.582 | | 0.148 | 1.71x10^-4^ | | 0.017 |
| rs17135355 | 6 | 2646011 | 22762 | | | | G | A | 0.250 | -0.573 | | 0.148 | 2.14x10^-4^ | | 0.019 |
| rs718603 | 6 | 2644011 | 20762 | | | | T | C | 0.250 | -0.573 | | 0.148 | 2.14x10^-4^ | | 0.019 |
| rs12526698 | 6 | 2665739 | 42490 | | | | G | T | 0.417 | 0.478 | | 0.133 | 5.53x10^-4^ | | 0.047 |
| **cg05673431 (*KSR1*)** | | | | | | | | | | | | | | | |
| rs6505279 | 17 | 27454868 | -89610 | | | | T | C | 0.339 | -0.553 | | 0.131 | 5.95x10^-5^ | | 0.026 |
| rs4795676 | 17 | 27447568 | -96910 | | | | A | G | 0.344 | -0.545 | | 0.132 | 8.94x10^-5^ | | 0.026 |
| rs4795677 | 17 | 27449636 | -94842 | | | | C | T | 0.344 | -0.545 | | 0.132 | 8.94x10^-5^ | | 0.026 |
| rs4795678 | 17 | 27462716 | -81762 | | | | C | T | 0.349 | -0.520 | | 0.131 | 1.47x10^-4^ | | 0.026 |
| rs6505292 | 17 | 27463695 | -80783 | | | | T | G | 0.349 | -0.520 | | 0.131 | 1.47x10^-4^ | | 0.026 |
| rs7210693 | 17 | 27463355 | -81123 | | | | G | A | 0.349 | -0.520 | | 0.131 | 1.47x10^-4^ | | 0.026 |
| *Genomic positions are indicated in hg38/GRCh38 Genome Assembly. meQTL: methylation quantitative trait loci; rsID: reference SNP cluster ID; Chr: chromosome; A1: effect allele; A2: non-effect allele; MAF: minor allele frequency (effect allele); SE: standard error; FDR: false discovery rate. | | | | | | | | | | | | | | | |
| **Table S9.** Summary results of in silico eQTL analyses. | | | | | | | | | | | | | | | |
| **rsID** | **Chr** | **Position** | | **A1** | **A2** | **Tissue** | | | **Gene** | | **Normalized effect size** | | | ***p*-value** | |
| rs11038897 | 11 | 46449277 | | T | C | Fibroblasts | | | *ATG13* | | -0.18 | | | 6.30x10^-14^ | |
| rs11038897 | 11 | 46449277 | | T | C | Lung | | | *MDK* | | -0.21 | | | 1.60x10^-8^ | |
| rs11038897 | 11 | 46449277 | | T | C | Lung | | | *ATG13* | | -0.17 | | | 2.60x10^-6^ | |
| rs11038897 | 11 | 46449277 | | T | C | Fibroblasts | | | *MADD* | | 0.14 | | | 6.50x10^-5^ | |
| rs11038897 | 11 | 46449277 | | T | C | Lung | | | *C11orf49* | | -0.12 | | | 1.40x10^-4^ | |
| rs11038897 | 11 | 46449277 | | T | C | Fibroblasts | | | *C11orf49* | | -0.13 | | | 4.10x10^-4^ | |
| rs6901966 | 6 | 2623299 | | C | G | Fibroblasts | | | *LINC01600* | | 0.24 | | | 3.90x10^-6^ | |
| rs6505279 | 17 | 27454868 | | T | C | Lung | | | *KSR1* | | -0.11 | | | 6.60x10^-5^ | |
| *Genomic positions are indicated in hg38/GRCh38 Genome Assembly. eQTL: expression quantitative trait loci; rsID: reference SNP cluster ID; Chr: chromosome; A1: effect allele; A2: non-effect allele. | | | | | | | | | | | | | | | |

| **Table S10.** Summary results of the eQTM analyses. | | | | | |
| --- | --- | --- | --- | --- | --- |
| **CpG** | **Gene** | **Coefficient** | **SE** | ***p*-value** | **FDR*** |
| cg10290200 | *FLNC* | -0.42 | 0.12 | 0.001 | 0.009 |
| cg10290200 | *ATP6V1F* | -0.19 | 0.14 | 0.175 | 0.647 |
| cg10290200 | *CALU* | -0.15 | 0.13 | 0.261 | 0.647 |
| cg10290200 | *KCP* | -0.14 | 0.14 | 0.303 | 0.647 |
| cg10290200 | *CCDC136* | 0.13 | 0.14 | 0.359 | 0.647 |
| cg10290200 | *IRF5* | 0.08 | 0.12 | 0.531 | 0.713 |
| cg10290200 | *ATP6V1FNB* | -0.08 | 0.13 | 0.554 | 0.713 |
| cg10290200 | *FAM71F2* | 0.05 | 0.14 | 0.704 | 0.792 |
| cg10290200 | *TNPO3* | 0.00 | 0.13 | 0.991 | 0.991 |
| cg05673431 | *KSR1* | -0.30 | 0.11 | 0.010 | 0.029 |
| cg05673431 | *LGALS9* | 0.27 | 0.12 | 0.026 | 0.039 |
| cg05673431 | *WSB1* | -0.16 | 0.12 | 0.176 | 0.176 |
| cg23032799 | *MDK* | 0.27 | 0.12 | 0.027 | 0.161 |
| cg23032799 | *AMBRA1* | 0.15 | 0.13 | 0.230 | 0.608 |
| cg23032799 | *CREB3L1* | -0.11 | 0.11 | 0.337 | 0.608 |
| cg23032799 | *CHRM4* | 0.10 | 0.12 | 0.405 | 0.608 |
| cg23032799 | *PHF21A* | 0.06 | 0.13 | 0.640 | 0.768 |
| cg23032799 | *DGKZ* | 0.03 | 0.12 | 0.783 | 0.783 |
| cg24254891 | *FAM83F* | -0.22 | 0.11 | 0.042 | 0.127 |
| cg24254891 | *TNRC6B* | 0.08 | 0.11 | 0.455 | 0.682 |
| cg24254891 | *GRAP2* | 0.02 | 0.11 | 0.888 | 0.888 |
| cg03629778 | *UBE2H* | -0.19 | 0.10 | 0.060 | 0.302 |
| cg03629778 | *ZC3HC1* | 0.15 | 0.11 | 0.157 | 0.393 |
| cg03629778 | *CPA4* | 0.06 | 0.11 | 0.600 | 0.895 |
| cg03629778 | *TMEM209* | -0.01 | 0.10 | 0.891 | 0.895 |
| cg03629778 | *KLHDC10* | 0.01 | 0.10 | 0.895 | 0.895 |
| cg16519100 | *SH3GL3* | -0.19 | 0.10 | 0.059 | 0.472 |
| cg16519100 | *RAMAC* | -0.13 | 0.11 | 0.208 | 0.768 |
| cg16519100 | *HDGFL3* | -0.10 | 0.10 | 0.345 | 0.768 |
| cg16519100 | *BTBD1* | -0.06 | 0.11 | 0.547 | 0.768 |
| cg16519100 | *BNC1* | 0.05 | 0.11 | 0.623 | 0.768 |
| cg16519100 | *HOMER2* | 0.05 | 0.11 | 0.659 | 0.768 |
| cg16519100 | *TM6SF1* | 0.04 | 0.11 | 0.695 | 0.768 |
| cg16519100 | *C15orf40* | -0.03 | 0.10 | 0.768 | 0.768 |
| cg12510044 | *PPM1F* | 0.19 | 0.12 | 0.134 | 0.413 |
| cg12510044 | *SDF2L1* | 0.18 | 0.12 | 0.159 | 0.413 |
| cg12510044 | *MAPK1* | 0.17 | 0.12 | 0.178 | 0.413 |
| cg12510044 | *YDJC* | 0.17 | 0.13 | 0.184 | 0.413 |
| cg12510044 | *TOP3B* | 0.08 | 0.13 | 0.502 | 0.904 |
| cg12510044 | *RIMBP3C* | -0.04 | 0.13 | 0.774 | 0.912 |
| cg12510044 | *PPIL2* | 0.02 | 0.13 | 0.879 | 0.912 |
| cg12510044 | *UBE2L3* | -0.01 | 0.12 | 0.904 | 0.912 |
| cg12510044 | *YPEL1* | 0.01 | 0.13 | 0.912 | 0.912 |
| cg26162522 | *PRKAA2* | -0.21 | 0.11 | 0.064 | 0.255 |
| cg26162522 | *FYB2* | 0.17 | 0.13 | 0.178 | 0.356 |
| cg26162522 | *RP1-158P9.3* | 0.06 | 0.13 | 0.665 | 0.805 |
| cg26162522 | *PLPP3* | -0.03 | 0.12 | 0.805 | 0.805 |
| cg16032470 | *BPIFB1* | -0.18 | 0.10 | 0.076 | 0.257 |
| cg16032470 | *CDK5RAP1* | -0.20 | 0.12 | 0.082 | 0.257 |
| cg16032470 | *BPIFB4* | 0.18 | 0.11 | 0.110 | 0.257 |
| cg16032470 | *SNTA1* | 0.09 | 0.12 | 0.451 | 0.649 |
| cg16032470 | *BPIFA2* | -0.08 | 0.11 | 0.464 | 0.649 |
| cg16032470 | *BPIFA1* | -0.05 | 0.11 | 0.683 | 0.797 |
| cg16032470 | *BPIFB2* | -0.02 | 0.11 | 0.826 | 0.826 |
| cg15833511 | *NDRG3* | 0.18 | 0.11 | 0.116 | 0.622 |
| cg15833511 | *MYL9* | 0.16 | 0.11 | 0.145 | 0.622 |
| cg15833511 | *RAB5IF* | 0.12 | 0.12 | 0.302 | 0.622 |
| cg15833511 | *SLA2* | 0.13 | 0.12 | 0.305 | 0.622 |
| cg15833511 | *TGIF2-RAB5IF* | 0.11 | 0.12 | 0.374 | 0.622 |
| cg15833511 | *TGIF2* | 0.10 | 0.12 | 0.402 | 0.622 |
| cg15833511 | *SOGA1* | 0.09 | 0.11 | 0.436 | 0.622 |
| cg15833511 | *DSN1* | -0.08 | 0.12 | 0.520 | 0.651 |
| cg15833511 | *DLGAP4* | -0.03 | 0.12 | 0.785 | 0.804 |
| cg15833511 | *TLDC2* | -0.03 | 0.13 | 0.804 | 0.804 |
| cg12845808 | *NDFIP1* | -0.17 | 0.11 | 0.125 | 0.327 |
| cg12845808 | *RNF14* | 0.15 | 0.11 | 0.193 | 0.327 |
| cg12845808 | *PCDH1* | -0.14 | 0.11 | 0.196 | 0.327 |
| cg12845808 | *GNPDA1* | -0.08 | 0.11 | 0.482 | 0.603 |
| cg12845808 | *DELE1* | 0.04 | 0.12 | 0.704 | 0.704 |
| cg03946667 | *SMAD5* | 0.16 | 0.12 | 0.166 | 0.332 |
| cg03946667 | *TGFBI* | -0.07 | 0.11 | 0.510 | 0.510 |
| cg10310427 | *DHX16* | 0.16 | 0.12 | 0.168 | 0.781 |
| cg10310427 | *GTF2H4* | -0.16 | 0.13 | 0.208 | 0.781 |
| cg10310427 | *FLOT1* | 0.14 | 0.13 | 0.269 | 0.781 |
| cg10310427 | *ATAT1* | -0.11 | 0.13 | 0.393 | 0.781 |
| cg10310427 | *GNL1* | 0.10 | 0.12 | 0.418 | 0.781 |
| cg10310427 | *ABCF1* | 0.09 | 0.12 | 0.457 | 0.781 |
| cg10310427 | *DDR1* | 0.09 | 0.12 | 0.463 | 0.781 |
| cg10310427 | *MDC1* | 0.09 | 0.12 | 0.473 | 0.781 |
| cg10310427 | *MUC21* | -0.08 | 0.12 | 0.480 | 0.781 |
| cg10310427 | *PRR3* | -0.08 | 0.11 | 0.491 | 0.781 |
| cg10310427 | *VARS2* | 0.08 | 0.13 | 0.546 | 0.781 |
| cg10310427 | *NRM* | 0.08 | 0.13 | 0.552 | 0.781 |
| cg10310427 | *C6orf136* | -0.07 | 0.13 | 0.606 | 0.793 |
| cg10310427 | *TUBB* | 0.05 | 0.12 | 0.700 | 0.850 |
| cg10310427 | *PPP1R10* | -0.03 | 0.13 | 0.807 | 0.907 |
| cg10310427 | *IER3* | -0.02 | 0.13 | 0.901 | 0.907 |
| cg10310427 | *PPP1R18* | 0.01 | 0.13 | 0.907 | 0.907 |
| cg00483640 | *MYLK4* | 0.15 | 0.12 | 0.228 | 0.349 |
| cg00483640 | *SERPINB1* | 0.12 | 0.12 | 0.322 | 0.349 |
| cg00483640 | *WRNIP1* | 0.12 | 0.13 | 0.349 | 0.349 |
| cg11023970 | *PRKCQ* | -0.12 | 0.11 | 0.280 | 0.487 |
| cg11023970 | *PFKFB3* | 0.08 | 0.11 | 0.451 | 0.487 |
| cg11023970 | *RBM17* | -0.07 | 0.10 | 0.487 | 0.487 |
| cg08161666 | *RASSF10* | -0.10 | 0.10 | 0.326 | 0.599 |
| cg08161666 | *TEAD1* | -0.06 | 0.11 | 0.599 | 0.599 |
| cg02424494 | *KLHL30* | -0.10 | 0.12 | 0.412 | 0.870 |
| cg02424494 | *ESPNL* | -0.08 | 0.12 | 0.510 | 0.870 |
| cg02424494 | *HES6* | -0.06 | 0.10 | 0.533 | 0.870 |
| cg02424494 | *TRAF3IP1* | 0.07 | 0.11 | 0.560 | 0.870 |
| cg02424494 | *ILKAP* | 0.06 | 0.12 | 0.607 | 0.870 |
| cg02424494 | *SCLY* | 0.05 | 0.12 | 0.665 | 0.870 |
| cg02424494 | *UBE2F-SCLY* | -0.05 | 0.12 | 0.676 | 0.870 |
| cg02424494 | *PER2* | 0.03 | 0.11 | 0.818 | 0.907 |
| cg02424494 | *ASB1* | -0.01 | 0.12 | 0.907 | 0.907 |
| cg12845391 | *IFNGR1* | -0.07 | 0.11 | 0.519 | 0.677 |
| cg12845391 | *IL20RA* | 0.05 | 0.11 | 0.677 | 0.677 |
| cg03956296 | *TMEM178A* | -0.04 | 0.10 | 0.686 | 0.846 |
| cg03956296 | *THUMPD2* | -0.02 | 0.11 | 0.846 | 0.846 |
| cg16769649 | *TNFRSF21* | -0.07 | 0.11 | 0.545 | 0.891 |
| cg16769649 | *CD2AP* | 0.01 | 0.11 | 0.891 | 0.891 |
| cg22208174 | *ZNF462* | 0.01 | 0.11 | 0.944 | 0.944 |
| *Adjusted *p*-value considering the number of tests carried out for each CpG site. SE: standard error; FDR: false discovery rate. | | | | | |

| **Table S11.** Summary findings of the enrichment gene-set analysis. | | | | | | |
| --- | --- | --- | --- | --- | --- | --- |
|  | **Cutoff**  ***p*<5x10^-4^** | | | **Cutoff *p*<5x10^-3^** | **Cutoff *p*<5x10^-5^** |  |
| **Term** | **OR** | ***p*-value** | ***q*-value** | ***p*-value** | ***p*-value** | **Genes** |
| **Biological Pathways (Bioplanet 2019)** | | | | | | |
| Interleukin-2 signaling pathway | 2.04 | 8.95x10^-7^ | 0.001 | 2.15x10^-9^ | 2.76x10^-4^ | *TES; MTCL1; CLTC; PRF1; RORA; ADRA1D; SLA2; ETS1; ETS2; CCND1; PDE4B; FAM53B; CCR6; CTSC; IER3; DUSP4; STARD5; VPS13A; TBC1D8; ANK2; BPGM; PPP2R5C; DUSP6; ITPKB; IRF4; GARS; EVL; IL6ST; VAMP5; MT1E; B4GALT5; HDAC4; MACF1; ENPEP; PFKFB3; PTGER3; PIK3R1; FOXO1; SOCS2; PAK1; MAT2A; RNF139; ATXN7; SPOCK2; PTK2B; ZSWIM6; E2F3; H1FX; KLRC1; NOP56; JUN; PTPRN2; PTCH1; GAD2; PER2; NR4A2; UCK2; KLF6; ID2; BHLHE40; YPEL1; KRAS; ABCE1; IL18R1* |
| Prolactin regulation of apoptosis | 3.81 | 1.99x10^-5^ | 0.010 | 7.23x10^-5^ | 0.001 | *CLIC6; DUSP4; TENM2; EGLN3; SSR3; BHLHA15; DUSP6; SOCS2; KLF6; CCND1; FTH1; TNS4; TRIB1; IER3; GET4; RALGPS2* |
| **Biological Pathways (Molecular Signatures Database)** | | | | | | |
| TNF-α Signaling via NF-kB | 4.00 | 8.65x10^-9^ | 4.33x10^-7^ | 2.27x10^-9^ | 3.04x10^-5^ | *PFKFB3; B4GALT1; TNFAIP2; ETS2; CCND1; PDE4B; PDLIM5; IER5; ICOSLG; IER3; DUSP4; KDM6B; JUN; DUSP2; SPHK1; INHBA; KLF4; NR4A2; SPSB1; KLF6; ID2; BHLHE40; F2RL1; SIK1; TRIB1; PLPP3; IL6ST; B4GALT5* |
| Hypoxia | 3.01 | 1.89x10^-5^ | 4.73x10^-4^ | 4.39x10^-4^ | 0.001 | *CDKN1C; CA12; ERRFI1; JUN; TES; PFKFB3; NEDD4L; RORA; AK4; ETS1; NDRG1; EXT1; KLF6; NOCT; BHLHE40; GCNT2; TGFBI; VHL; PCK1; PPARGC1A; IER3; MT1E* |
| IL-2/STAT5 Signaling | 2.56 | 4.36x10^-4^ | 0.006 | 1.50x10^-5^ | 0.047 | *CDKN1C; POU2F1; IFNGR1; CD81; PTCH1; CAPG; RORA; NDRG1; ETV4; SOCS2; UCK2; MUC1; KLF6; IRF4; BHLHE40; COL6A1; IL18R1; TNFRSF21; WLS* |
| Estrogen Response Late | 2.55 | 4.64x10^-4^ | 0.006 | 0.001 | 0.048 | *CHST8; CA12; DUSP2; ITPK1; PTGER3; EMP2; KLF4; TOB1; AFF1; ASS1; SCUBE2; CCND1; IMPA2; ID2; DLG5; RBBP8; IL6ST; S100A9; LLGL2* |
| Hedgehog Signaling | 4.81 | 0.003 | 0.025 | 0.002 | 0.049 | *TLE3; HEY1; DPYSL2; PTCH1; NF1; ETS2* |
| Apoptosis | 2.30 | 0.005 | 0.039 | 7.98x10^-5^ | 0.005 | *JUN; ANXA1; IFNGR1; SLC20A1; MMP2; F2R; PRF1; HSPB1; PAK1; KRT18; CCND1; VDAC2; CTNNB1; IER3* |
| **Gene Ontologies (GO): Biological Process 2019** | | | | | | |
| Negative regulation of cell population proliferation | 2.42 | 1.12x10^-5^ | 0.039 | 2.85x10^-12^ | 0.004 | *CDKN1C; CEBPA; TES; CTBP1; ETS1; TOB1; PAK1; ACVR1C; RNF139; FTH1; PTK2B; E2F3; SOX9; VHL; CDKN2B; CTNNBIP1; F2R; PROX1; INHBA; PPP2R5C; KLF4; RUNX3; MTSS1; MYO16; PARP10; PER2; RBP4; SFRP2; WNK2; DLG5; NF1; CTNNB1; TRIB1; HSPA1B* |
| **Gene Ontologies (GO): Cellular Components 2019** | | | | | | |
| Actin cytoskeleton | 2.47 | 3.23x10^-5^ | 0.010 | 4.14x10^-5^ | 0.013 | *MACF1; TMEM63B; AVIL; ABCB4; IPP; FAM107A; CAPG; MYLK3; NEURL1B; PAK1; SIPA1L1; SH3PXD2B; KNTC1; PDLIM5; MARK2; NDC1; ACTR2; CARMIL2; ANXA1; RDX; PAWR; DCDC2; COBL; BAIAP2; MTSS1; FER; ARPC3; ZNF74; LLGL2* |
| **Protein-Protein Interactions Hub dataset (Proteins)** | | | | | | |
| GSK3B | 1.95 | 2.68x10^-5^ | 0.009 | 4.23x10^-7^ | 0.021 | *CTNND2; ITSN1; LPCAT1; JPH1; ETS1; AFF1; GOLGA2; ZMYM2; CCND1; CREB3L1; DPYSL2; UIMC1; PDE4B; LBR; IER3; BRD2; TLE3; IFNGR1; COBL; ANK2; SYN3; MAPK8IP1; GEMIN4; ARHGEF2; PLCB1; KCTD15; HDAC4; CEBPA; MACF1; SLC20A1; FOXO1; PAK1; MUC1; EPB41L1; ATXN7; FADD; VHL; PPARGC1A; MARK2; SPEN; JUN; GABBR1; FUS; NFATC4; ZC3HC1; KLF5; CABYR; ID2; SNAI1; CTNNB1; DCP1A* |
| MAPK1 | 2.09 | 2.34x10^-4^ | 0.021 | 2.76x10^-5^ | 0.007 | *HDAC4; CEBPA; THRB; TWIST1; RORA; PLA2G6; ETS1; TOB1; FOXO1; ETS2; PAK1; ZMYM2; PDE4B; IER3; DUSP4; JUN; DUSP2; KSR1; KCNJ11; SPHK1; PDE4D; MKL1; BRAF; PPP2R5C; DUSP6; NFATC4; GJD2; NR4A2; ID2; MAP3K10; ARHGEF2; PLCB1* |
| MAPK9 | 2.54 | 2.53x10^-4^ | 0.021 | 8.96x10^-5^ | 0.002 | *DUSP4; MACF1; JUN; DUSP2; ITSN1; DUSP19; WDR62; SYN3; TOB1; DUSP6; ETS2; MAPK8IP1; NFATC4; CCND1; CTNNB1; MAP3K10; FADD; KCTD15; JDP2; HSPA1B; MARK2* |
| MAPK3 | 2.20 | 3.24x10^-4^ | 0.022 | 8.54x10^-5^ | 0.005 | *HDAC4; CEBPA; THRB; TWIST1; PLA2G6; ETS1; TOB1; ETS2; ZFP36L1; PPARGC1A; IER3; DUSP4; JUN; DUSP2; KSR1; SPHK1; PDE4D; MKL1; BRAF; ETV1; SYN3; DUSP6; NFATC4; GJD2; ID2; ARHGEF2; PLCB1* |
| PRKACA | 1.98 | 4.59x10^-4^ | 0.026 | 3.58x10^-6^ | 0.004 | *PFKFB3; CLTC; NEDD4L; HSPB1; NDRG1; CYP17A1; PAK1; CCND1; DPYSL2; PDE4B; SOX9; PPARGC1A; TEAD1; ANXA1; POU2F1; KSR1; KCNJ11; PDE4D; EN2; GAD2; BRAF; ANK2; ETV1; GJD2; ITPKB; PRKAR1A; HAND2; KCNQ1; SNAI1; SIK3; CTNNB1; ARHGEF2; PLCB1* |
| **Protein-Protein Interactions dataset (Transcription Factors)** | | | | | | |
| EP300 | 2.08 | 9.16x10^-5^ | 0.021 | 2.54x10^-10^ | 0.045 | *CEBPA; KDM1A; CTBP1; CLTC; TWIST1; RORA; PIK3R1; ETS1; FOXO1; ETS2; HDAC7; GOLGA2; CCND1; ATXN7; CREB3L1; EPC1; SOX9; VHL; KPNA2; PCK1; PPARGC1A; JUN; WDR59; SPHK1; ARNT; ETV1; PROX1; PPP2R5C; KLF4; RUNX3; ETV4; KLF5; KRT18; HAND2; CTNNB1; JDP2; CAMK1G* |
| ESR1 | 1.73 | 2.03x10^-4^ | 0.021 | 2.66x10^-7^ | 0.013 | *THRB; KDM1A; CHD9; CLTC; HSPB1; RPL8; SYNE1; GOLGA2; PSMD6; CCND1; OPA1; SNRPD1; UIMC1; TRIM24; RRP12; SCYL2; VAV3; ACTR2; DDX18; MRPS22; SSR3; ARNT; WIPI1; MRPS9; GARS; CD47; GLYR1; HDAC4; CEBPA; RPN2; PIK3R1; FOXO1; HDAC7; PRDX2; PAK1; MUC1; MYH14; FLNC; PPARGC1A; PPARGC1B; MARK2; NOP56; DYNC1H1; FIS1; JUN; KDM4B; POU2F1; FUS; RDX; RRP1B; FAM213A; POU4F1; KLF5; KRT18; SPAG1; CTNNB1; HSPA1B* |
| SMAD3 | 2.12 | 2.81x10^-4^ | 0.021 | 8.60x10^-7^ | 0.033 | *HDAC4; CEBPA; CHRD; NEDD4L; JPH1; ETS1; TOB1; FOXO1; ZMYM2; ACVR1C; HEY1; OPA1; RIOK2; SOX9; PITX1; KDM6B; JUN; MKL1; RPA1; HMGA2; PROX1; KLF4; RUNX3; BMP7; KLF5; SIK3; SNAI1; NF1; CTNNB1; HSPA1B* |
| **Drug Signature Database (DSigDB)*** | | | | | | |
| Estradiol  CTD 00005920 | 1.77 | 5.78x10^-13^ | 1.08x10^-9^ | 1.97x10^-20^ | 2.73x10^-4^ | *ERRFI1; LPGAT1; TES; TRIO; RPL34; FZD10; PSMD6; BPIFA1; ZNF608; FTH1; SMCO4; SOX9; LGALS8; IER5; WLS; IER3; SULT2A1; CDK5RAP2; CDON; MBNL1; STARD5; ACOT7; FNBP1; BHLHA15; CMBL; RUNX3; RBP4; PRR16; TRIB1; MT1E; LLGL2; ATP5S; CDS1; MACF1; CTBP1; ABCB4; LPAR1; NEDD4L; TWIST1; TMTC1; NCAPH; PRDX2; EPB41L1; C14ORF180; ZNF821; FADD; PCK1; PPARGC1A; ST3GAL1; PPARGC1B; EDEM3; JUN; BCL11A; FZD7; PARP12; NFATC4; EXT1; FERMT1; EHD2; UCK2; PPFIBP2; ID2; SNAI1; SSPN; FAT3; FARSA; MRAP2; CPEB2; CDKN1C; CEP57; CLIC6; KLLN; NCAPG2; STON2; JPH1; EPB41L4A; HEY1; ENC1; TRIM3; RPLP2; RRP12; CTSC; RALGPS2; CHST8; DUSP4; VAV3; BRD2; DUSP2; ANXA1; PRMT7; MME; SPHK1; ITPK1; PAWR; EMP2; PROX1; NBPF1; DUSP6; MLF1; MTSS1; PIGC; F7; PAX7; B3GNT6; GARS; ARHGEF2; C9ORF3; PLCB1; JDP2; SDCCAG8; C11ORF52; SLC20A1; PRUNE2; PTGER3; ADAM22; FIGN; HSD17B7; SCEL; PAK1; RNF139; TIAF1; GCNT2; ZNF469; FLNC; CD58; EXOSC2; CA12; EGLN3; GABBR1; CDKN2B; PLEKHA2; RFTN1; RRP1B; KRT18; SLCO3A1; F2RL1; CTNNB1; EBAG9; ADGRL2; HSPA1B; CSF3; STEAP3; CTNND2; FAM107A; RORA; ETS1; MYLK3; ETS2; FOXQ1; FADS2; DEPTOR; CCND1; GRB14; KPNA2; PITX1; NKX6-2; IGFBP2; ATP11B; TMEM134; ZNF91; RAB30; IL6ST; S100A9; MAD1L1; KLHL18; PFKFB3; ARHGEF26; CHRD; AK4; NRF1; NDRG1; FOXO1; ZFP36L1; SOCS2; SCUBE2; BRINP2; DPP7; PSAP; KLRC1; PRODH; CAV2; INHBA; DNAJC15; ARHGAP26; BMP7; LSM2; NR4A2; NAT1; DLG5; BHLHE40; NF1; RNF181; TGFBI; GCLM; CNIH3; B4GALT1; HSPB1; ADRA1D; AFF1; SYNE1; BOC; PDE4B; BDKRB1; UNKL; MSMB; ABCC4; ABCC1; KSR1; APAF1; SGIP1; MMP2; ARNT; COBL; ETV1; WIPI1; BPGM; ETV4; MUT; UPK3A; ZFP90; ASPH; POLR1A; DAD1; HAND2; COL6A1; MCM3; COL6A3; EVL; AGXT; TNFRSF21; HDAC4; CEBPA; SLC47A1; TUFT1; PRR5L; TTL; TOB1; HDAC7; MUC1; ACVR1C; MAT2A; GNG4; RBBP8; E2F3; IGF2BP2; PDLIM5; SLC25A22; NOP56; MBOAT1; CABLES1; BAIAP2; KLF4; ASS1; KBTBD11; SLC4A7; KLF6; KLF5; KRAS; SPRY1; CDO1* |
| Trichostatin A  CTD 00000660 | 1.82 | 8.76x10^-13^ | 1.08x10^-9^ | 7.08x10^-36^ | 0.001 | *ERRFI1; IPO11; LPGAT1; TES; TRIO; IPP; FZD10; SLCO6A1; ZNF608; DPYSL2; EPC1; SOX9; EPHB2; LGALS8; IER5; IER3; CDON; MBNL1; DDX11; ANK2; UNC5D; SFRP2; PRR16; FAM76B; SIK1; CAGE1; TRIB1; KCTD15; LLGL2; ATP5S; ZNF397; CDS1; ENPEP; CASZ1; CDCA7; IL20RB; NEDD4L; TWIST1; TMTC1; PIK3R1; EPB41L1; PCK1; PPARGC1A; JUN; WDR59; PTPRN2; BCL11A; PCDH20; ST8SIA2; FZD7; ST8SIA3; LARP7; BRAF; SLC16A14; NFATC4; DAZL; EXT1; FERMT1; STAG1; PPFIBP2; ID2; SSPN; FAT4; GPR19; CLIC6; HBS1L; LPCAT1; TMEM51; TFCP2L1; STON2; JPH1; EPB41L4A; SLC22A15; HEY1; CHL1; ENC1; FLVCR2; CCR6; TEAD1; CTSC; RALGPS2; DUSP4; VAV3; ANXA1; MME; SPHK1; SIRT4; PAWR; EMP2; PROX1; NBPF1; MLF1; MTSS1; CLDN10; PAX7; ARHGEF3; ARHGEF4; CD47; C9ORF3; MFGE8; PLCB1; VAMP5; SDCCAG8; C11ORF52; GRAMD1B; PRUNE2; PTGER3; FIGN; NEURL1B; EPCAM; CD58; GPR157; PURG; CA12; WWOX; EGLN3; CDKN2B; PLEKHA2; RFTN1; RPA1; POU4F1; PER2; PRPF39; KRT18; KLHL8; YPEL1; PCYOX1L; F2RL1; CTNNB1; STEAP3; RTKN; CTNND2; JADE1; RORA; MSI2; ETS1; FOXQ1; TMEM144; DEPTOR; CCND1; GRB14; SALL3; CWC22; PABPC4L; KCNH5; KCNH8; SEMA6D; F2R; RAB30; DOK5; IL6ST; GLYR1; AK4; DNAJC24; FOXO1; SOCS2; SCUBE2; ATXN7; RAB6B; OSBPL6; POU2F1; CAV2; CCDC136; PTCH1; HMGA2; ARHGAP26; BMP7; NR4A2; SEPSECS; GCLC; BHLHE40; NF1; LHX5; TGFBI; TTC23; THRB; CHD9; SYNE1; ELAVL3; TRPS1; BOC; UNKL; SCYL2; ACTR2; APAF1; MMP2; RFPL2; PDE4D; VPS13A; COBL; ETV1; PPP2R5C; RABGGTB; ZFP90; ADAMTS20; SENP1; DNM2; ASPH; ZNF516; COL6A1; MCM3; TNFRSF21; HDAC4; PRKAA2; PRR5L; HDAC7; ACVR1C; RBBP8; PDLIM5; LINGO2; KDM4B; B3GAT2; EYA4; MBOAT1; ASS1; KBTBD11; SLC4A7; KLF6; MEIS1; KLF5; SPAG1; SPRY1; SEC24D; CBLN1* |
| Benzo[a]pyrene  CTD 00005488 | 1.76 | 1.06x10^-12^ | 1.08x10^-9^ | 2.40x10^-30^ | 2.19x10^-5^ | *SPON2; IPO11; TRIO; SLC46A2; ITSN1; AQP7; FZD10; FNBP1L; NADSYN1; SOX21; SNRPD1; DPYSL2; FTH1; TRIM24; FAM53B; CHAC1; SOX9; LGALS8; TNS4; IER5; SIL1; ICOSLG; IER3; SULT2A1; CDK5RAP2; FNBP1; ANK2; IFT80; CMBL; RUNX3; LARS2; KCNQ1; ZNF710; TRIB1; MT1E; ZNF397; ABCB4; CEP85L; CDCA7; AGAP1; NEDD4L; TMTC1; NCAPH; ABR; PRDX2; EPB41L1; LDHD; VTI1A; LTA4H; PCK1; PPARGC1A; ST3GAL1; GTF2A1L; JUN; PTPRN2; BCL11A; FZD7; RDX; SLC35E3; EXT1; FERMT1; LACC1; STAG1; PPFIBP2; CABYR; GPR180; ID2; SNAI1; CPEB2; CDKN1C; TENM2; LPCAT1; PTPRM; JPH1; EPB41L4A; SIPA1L1; SLC22A18; ENC1; CCR6; TEAD1; NDC1; VAV3; DUSP2; ZNF362; MME; KCND3; ITPK1; EMP2; AP3B1; PROX1; DUSP6; CDYL2; MTSS1; F7; DMC1; ARHGEF3; GARS; C9ORF3; PLCB1; VAMP5; JDP2; SDCCAG8; OCA2; SLC20A1; PRUNE2; CAPG; NEURL1B; GCNT2; FLNC; CD58; ZNF589; CA12; ZNF462; WWOX; CDKN2B; PLEKHA2; RRP1B; POU4F1; SPSB1; CMSS1; KRT18; SLCO3A1; F2RL1; CTNNB1; IL18R1; HSPA1B; ALDH1L1; STEAP3; EXOC3L4; KDM1A; CTNND2; JADE1; RORA; MSI2; MYLK3; ETS2; FOXQ1; STK10; DEPTOR; CCND1; KNTC1; PDE8B; ZNF565; TLE3; KCNH5; DGAT1; CTNNBIP1; USP2; F2R; IGFBP2; ALG14; MR1; HIC1; GLTPD2; STIM2; LY6D; MAD1L1; GLYR1; CLEC16A; NRF1; NDRG1; FOXO1; SOCS2; SCUBE2; C1ORF105; ATXN7; KLRC1; PRODH; FAM83D; IVNS1ABP; POU2F1; FUS; CD160; ESRRB; DCDC2; HMGA2; FOXN3; DNAJC15; ARHGAP26; BMP7; ZC3HC1; GCLC; FER; NAT1; MAFG; NF1; GCLM; SERINC2; CNIH3; THRB; DOCK3; B4GALT1; MTCL1; CHD9; LRRK1; TNFAIP2; HSPB1; DUSP13; ELAVL3; TRPS1; PDE4B; EDIL3; UNKL; LBR; MSMB; MKL2; ARHGEF10; ABCC4; ABCC1; ADGRV1; PDCD6IP; APAF1; PDE4D; VPS13A; ARNT; COBL; PPP2R5C; RABGGTB; ETV4; UPK3A; GFOD1; ASPH; IRF4; MCM3; EVL; DNASE2; AGXT; PPP1R12B; TNFRSF21; B4GALT5; HDAC4; CEBPA; VAC14; RBM47; SEMA3D; TUFT1; SEMA3G; TOB1; ACVR1C; WNT11; MAT2A; GNG4; SPOCK2; IGF2BP3; PDLIM5; NOP56; KDM4B; GALNT2; KCNIP4; MBOAT1; BAIAP2; BICC1; ASS1; KBTBD11; SLC4A7; KLF6; KLF5; SPAG1; SPRY1; SLC25A31; SEC24D; CDO1; SNTB2* |
| *Only the top-three significant results are reported in this table. A full report including the 118 significant terms is reported in the Zenodo repository as described in the Data availability statement. OR: odds ratio. | | | | | | |

| **Table S12.** Summary results of DMRs identified with comb-p. | | | | | | | |
| --- | --- | --- | --- | --- | --- | --- | --- |
| **Gene** | **Chr** | **Start*** | **End*** | **Width** | **Nº CpGs** | **Region**  ***p*-value** | **Adjusted**  ***p*-value** |
| *GLTPD2* | 17 | 4788081 | 4788183 | 103 | 3 | 5.91x10^-16^ | 3.75x10^-12^ |
| *CREB3L1* | 11 | 46274469 | 46274643 | 175 | 4 | 2.48x10^-13^ | 9.81x10^-10^ |
| *MYLK4-LINC01600* | 6 | 2623249 | 2623545 | 297 | 2 | 8.76x10^-11^ | 2.04x10^-7^ |
| *CABYR* | 18 | 23992402 | 23992785 | 384 | 5 | 4.37x10^-8^ | 7.86x10^-5^ |
| *NDRG1* | 8 | 133298310 | 133298476 | 167 | 3 | 3.20x10^-8^ | 1.33x10^-4^ |
| *PER2* | 2 | 238300705 | 238300990 | 286 | 3 | 2.47x10^-7^ | 0.001 |
| *FOXQ1* | 6 | 1228715 | 1228752 | 38 | 2 | 3.61x10^-8^ | 0.001 |
| *LACC1* | 13 | 43879047 | 43879377 | 331 | 9 | 7.28x10^-7^ | 0.002 |
| *ETNPPL* | 4 | 108941832 | 108942157 | 326 | 2 | 1.11x10^-6^ | 0.002 |
| *CD93* | 20 | 23186897 | 23187039 | 143 | 2 | 4.89x10^-7^ | 0.002 |
| *TNIP1* | 5 | 151087127 | 151087279 | 153 | 4 | 9.06x10^-7^ | 0.004 |
| *BTBD3* | 20 | 11301674 | 11301938 | 265 | 3 | 1.32x10^-6^ | 0.003 |
| *KSR1* | 17 | 27544478 | 27544765 | 288 | 2 | 3.04x10^-6^ | 0.007 |
| *CSF3* | 17 | 40015202 | 40015445 | 244 | 5 | 3.27x10^-6^ | 0.009 |
| *DAZL* | 3 | 16605476 | 16605619 | 144 | 4 | 2.66x10^-6^ | 0.013 |
| *CCDC33* | 15 | 74236265 | 74236379 | 115 | 2 | 4.14x10^-6^ | 0.025 |
| *SCGB1A1* | 11 | 62396963 | 62396980 | 18 | 2 | 8.05x10^-7^ | 0.032 |
| *Genomic positions are indicated in hg38/GRCh38 Genome Assembly. DMR: differentially methylated region; Chr: chromosome. | | | | | | | |

| **Table S13.** Summary results of DMRs identified with DMRcate. | | | | | | |
| --- | --- | --- | --- | --- | --- | --- |
| **Gene** | **Chr** | **Start*** | **End*** | **Width** | **Nº CpGs** | **FDR** |
| *MYLK4-LINC01600* | 6 | 2623248 | 2623543 | 296 | 2 | 2.12x10^-12^ |
| *GLTPD2* | 17 | 4787501 | 4788181 | 681 | 5 | 2.12x10^-12^ |
| *CREB3L1-CTD-2589M5.4* | 11 | 46274468 | 46275243 | 776 | 6 | 6.32x10^-10^ |
| *MAPK1* | 22 | 21761183 | 21761384 | 202 | 2 | 3.31x10^-9^ |
| *PER2* | 2 | 238290900 | 238291710 | 811 | 3 | 1.97x10^-7^ |
| *KSR1* | 17 | 27544477 | 27544763 | 287 | 2 | 2.01x10^-6^ |
| *TTC39C* | 18 | 23992401 | 23992783 | 383 | 5 | 3.30x10^-6^ |
| *CTBP1* | 4 | 1230844 | 1231255 | 412 | 3 | 7.52x10^-6^ |
| *TGFBI* | 5 | 136058353 | 136059077 | 725 | 5 | 7.52x10^-6^ |
| *NDRG1* | 8 | 133298309 | 133298474 | 166 | 3 | 2.06x10^-4^ |
| PER2 | 2 | 238300704 | 238300774 | 71 | 2 | 4.83x10^-4^ |
| *FOXQ1* | 6 | 1228714 | 1228750 | 37 | 2 | 5.16x10^-4^ |
| *DAZL* | 3 | 16605396 | 16605617 | 222 | 5 | 8.02x10^-4^ |
| *Genomic positions are indicated in hg38/GRCh38 Genome Assembly. DMR: differentially methylated region; Chr: chromosome. | | | | | | |

| **Supplementary Table S14**. Summary table of heterogeneity assessment on stratified analyses by biological sex. | | | | | | | | | | | |
| --- | --- | --- | --- | --- | --- | --- | --- | --- | --- | --- | --- |
|  |  |  |  | **Females (n=46)** | | | **Males (n=51)** | | | **Meta-analysis** | |
| **CpG** | **Gene** | **Chr** | **Position*** | **logFC** | **SE** | ***p*** | **logFC** | **SE** | ***p*** | **I^2^** | **Q-*p*** |
| cg10290200 | *FLNC* | 7 | 128841241 | -0.48 | 0.07 | 1.17x10^-8^ | -0.32 | 0.05 | 1.09x10^-8^ | 0 | 0.335 |
| cg00483640 | *MYLK4-LINC01600* | 6 | 2623249 | -0.44 | 0.06 | 6.13x10^-10^ | -0.27 | 0.05 | 2.08x10^-6^ | 0 | 0.326 |
| cg15833511 | *SLA2* | 20 | 36628395 | -0.37 | 0.06 | 3.65x10^-8^ | -0.27 | 0.05 | 2.72x10^-7^ | 86.29 | 0.007 |
| cg24254891 | *FAM83F* | 22 | 39999311 | -0.33 | 0.05 | 2.17x10^-7^ | -0.26 | 0.05 | 2.37x10^-6^ | 0 | 0.600 |
| cg12510044 | *MAPK1* | 22 | 21761184 | -0.35 | 0.06 | 3.05x10^-7^ | -0.20 | 0.04 | 8.07x10^-6^ | 0 | 0.599 |
| cg02424494 | *PER2* | 2 | 238291487 | -0.29 | 0.04 | 1.79x10^-8^ | -0.15 | 0.04 | 8.20x10^-4^ | 47.35 | 0.168 |
| cg03629778 | *ZC3HC1* | 7 | 130051485 | -0.20 | 0.04 | 1.06x10^-5^ | -0.20 | 0.04 | 1.01x10^-5^ | 0 | 0.543 |
| cg05673431 | *KSR1* | 17 | 27544478 | -0.23 | 0.04 | 3.99x10^-7^ | -0.12 | 0.03 | 4.52x10^-4^ | 0 | 0.794 |
| cg11023970 | *PFKFB3* | 10 | 6295886 | 0.20 | 0.04 | 2.43x10^-5^ | 0.18 | 0.03 | 9.03x10^-7^ | 39.81 | 0.197 |
| cg03956296 | *THUMPD2* | 2 | 39724059 | -0.40 | 0.10 | 3.61x10^-4^ | -0.54 | 0.10 | 1.61x10^-6^ | 46.03 | 0.173 |
| cg23032799 | *CREB3L1* | 11 | 46274563 | -0.20 | 0.04 | 4.33x10^-5^ | -0.15 | 0.04 | 1.69x10^-4^ | 71.57 | 0.061 |
| cg08161666 | *ARNTL* | 11 | 13013350 | -0.32 | 0.10 | 0.002 | -0.40 | 0.06 | 6.62x10^-8^ | 0 | 0.634 |
| cg10310427 | *IER3* | 6 | 30749208 | -0.23 | 0.05 | 1.81x10^-5^ | -0.12 | 0.03 | 1.97x10^-4^ | 28.13 | 0.238 |
| cg16769649 | *TNFRSF21* | 6 | 47358617 | -0.37 | 0.07 | 1.35x10^-6^ | -0.18 | 0.05 | 0.001 | 0 | 0.590 |
| cg26162522 | *PPAP2B* | 1 | 56571443 | -0.31 | 0.05 | 8.61x10^-8^ | -0.10 | 0.04 | 0.022 | 0 | 0.730 |
| cg16032470 | *BPIFA1* | 20 | 33230393 | -0.17 | 0.04 | 2.34x10^-4^ | -0.17 | 0.04 | 5.39x10^-5^ | 69.79 | 0.069 |
| cg03068616 | *LY86* | 6 | 6678265 | -0.26 | 0.06 | 2.02x10^-4^ | -0.28 | 0.06 | 1.39x10^-5^ | 4.62 | 0.306 |
| cg16519100 | *HDGFRP3* | 15 | 83200514 | 0.34 | 0.06 | 2.48x10^-7^ | 0.20 | 0.06 | 0.003 | 40.92 | 0.193 |
| cg22208174 | *ZNF462* | 9 | 106880861 | -0.22 | 0.06 | 0.001 | -0.25 | 0.05 | 8.11x10^-6^ | 0 | 0.394 |
| cg12845391 | *IFNGR1* | 6 | 137224161 | -0.13 | 0.04 | 8.70x10^-4^ | -0.12 | 0.03 | 6.97x10^-5^ | 0 | 0.706 |
| cg12845808 | *PCDH12* | 5 | 141959039 | -0.12 | 0.03 | 1.13x10^-4^ | -0.10 | 0.03 | 6.04x10^-4^ | 12.32 | 0.286 |
| cg03946667 | *TGFBI* | 5 | 136058672 | -0.20 | 0.04 | 2.77x10^-6^ | -0.11 | 0.03 | 0.003 | 35.49 | 0.213 |
| *Genomic positions are indicated in hg38/GRCh38 Genome Assembly. Chr: chromosome; logFC: log_2_(fold-change); SE: standard error; Q-P: Cochran's Q *p*-value. | | | | | | | | | | | |
